# Methods for studying microbial acid stress responses: from molecules to populations

**DOI:** 10.1093/femsre/fuae015

**Published:** 2024-05-17

**Authors:** Merve Atasoy, Simona Bartkova, Zeynep Çetecioğlu-Gürol, Nuno P Mira, Conor O'Byrne, Fernando Pérez-Rodríguez, Aricia Possas, Ott Scheler, Jana Sedláková-Kaduková, Mirka Sinčák, Matthias Steiger, Carmit Ziv, Peter A Lund

**Affiliations:** UNLOCK, Wageningen University and Research, PO Box 9101, 6700 HB, the Netherlands; Department of Chemistry and Biotechnology, Tallinn University of Technology, Ehitajate tee 5, 19086 Tallinn, Estonia; Department of Industrial Biotechnology, KTH Royal Institute of Technology, Roslagstullsbacken 21 106 91 Stockholm, Stockholm, Sweden; iBB, Institute for Bioengineering and Biosciences, Department of Bioengineering, Universidade de Lisboa, Av. Rovisco Pais, 1049-001 Lisboa, Portugal; Associate Laboratory i4HB, Institute for Health and Bioeconomy, Instituto Superior Técnico, Universidade de Lisboa, Av. Rovisco Pais, 1049-001 Lisboa, Portugal; Microbiology, School of Biological and Chemical Sciences, University of Galway, University Road, Galway, H91 TK33, Ireland; Department of Food Science and Tehcnology, UIC Zoonosis y Enfermedades Emergentes ENZOEM, University of Córdoba, 14014 Córdoba, Spain; Department of Food Science and Tehcnology, UIC Zoonosis y Enfermedades Emergentes ENZOEM, University of Córdoba, 14014 Córdoba, Spain; Department of Chemistry and Biotechnology, Tallinn University of Technology, Ehitajate tee 5, 19086 Tallinn, Estonia; Institute of Chemistry and Environmental Sciences, University of Ss. Cyril and Methodius, 91701 Trnava, Republic of Slovakia; Institute of Chemistry and Environmental Sciences, University of Ss. Cyril and Methodius, 91701 Trnava, Republic of Slovakia; Institute of Chemical, Environmental and Bioscience Engineering, TU Wien, Getreidemarkt 9, 1060 Vienna, Austria; Department of Postharvest Science, Agricultural Research Organization, Volcani Center, 7505101 Rishon LeZion, Israel; School of Biosciences and Institute of Microbiology of Infection, University of Birmingham, Birmingham B15 2TT, United Kingdom

**Keywords:** acid resistance, acid stress responses, microbiological methods, acid tolerance

## Abstract

The study of how micro-organisms detect and respond to different stresses has a long history of producing fundamental biological insights while being simultaneously of significance in many applied microbiological fields including infection, food and drink manufacture, and industrial and environmental biotechnology. This is well-illustrated by the large body of work on acid stress. Numerous different methods have been used to understand the impacts of low pH on growth and survival of micro-organisms, ranging from studies of single cells to large and heterogeneous populations, from the molecular or biophysical to the computational, and from well-understood model organisms to poorly defined and complex microbial consortia. Much is to be gained from an increased general awareness of these methods, and so the present review looks at examples of the different methods that have been used to study acid resistance, acid tolerance, and acid stress responses, and the insights they can lead to, as well as some of the problems involved in using them. We hope this will be of interest both within and well beyond the acid stress research community.

## Teaser

This review covers the main methods that are used to study microbial acid stress responses.

## Introduction

Micro-organisms are the most successful and ubiquitous colonizers of the diverse environments of the biosphere, where they can flourish with or without oxygen, at extremes of temperature, water availability, ionic strength, pressure, and pH. From our human perspective, we label many of these organisms as extremophiles and marvel at their ability to cope with conditions that would be instantly lethal for us. In practice, however, any conditions can be extreme to organisms that have not evolved to grow and reproduce in them. The oxygen we rely on is death to obligate anaerobes; extreme halophiles lyse in pure water; acidophiles isolated from acidic mine drainage with a pH of 3 cannot grow at pH 7 (Mohr and Larsen 1963, Wichlacz and Unz et al. 1981, Lu and Imlay 2021). And this in turn makes it all the more remarkable that many micro-organisms are able to tolerate significant changes in the conditions under which they survive or grow. This feat often requires the expression of new properties, switched on by exposure to these altered conditions, a phenomenon called a stress response.

There are many reasons to study stress resistance and the ways in which micro-organisms can respond to become more resistant to stresses in their external environments. The study of how micro-organisms adapt in the short term to changing environments while still having to operate within physical and chemical constraints has led to innumerable insights in fields as diverse as cellular physiology, signal transduction, gene regulation, membrane biochemistry, and protein folding. These findings also have many applications in areas including infection, food microbiology, and biotechnology. For example, stress responses pose problems in product safety, since micro-organisms can use them to quickly adapt to the conditions that manufacturers are using to try to kill or inactivate them (Chen 2017). Conversely, if metabolism (innate or engineered) is being exploited to develop new manufacturing processes, understanding how micro-organisms can adapt to unfavourable conditions can help the design of organisms with improved growth or higher product yields. And understanding how micro-organisms respond to the stresses that they are subjected to from the immune system, or in our attempts to kill them with antibiotics, antifungals, and disinfectants, can lead to better understanding of infectious disease, including identifying targets for future drug design (Poole 2012).

Many different methods have been used in studying, understanding, and exploiting stress resistance and stress responses. Micro-organisms may be studied as single cells, or as pure cultures or components of simple or complex microbial consortia, grown under laboratory conditions or in simulated or real environments of relevance to their application, as planktonic or biofilm cultures or mixtures of the two. They may be assessed for viability, culturability, survival, growth, resilience, and dormancy, by methods ranging from simple plate counts to flow cytometry. The tools that are brought to bear may be biophysical, molecular, cellular, physiological, biochemical, classical genetic, genomic, or some combination of any or all of these. The difficulties of directly studying populations in complex environments such as within food, coupled with the need to make predictions about how they may behave in these environments, means that mathematical modelling is an additional important tool. This breadth of methodology means that it can be challenging for newcomers to enter the field, and there is also the risk of research teams becoming overspecialized, with different areas of research becoming more separated, rather than overlapping and cross-fertilizing each other.

With all the above points in mind, the COST Action CA18113 ‘Understanding and exploiting the impacts of low pH on micro-organisms’ was launched in 2019, with the aim of ‘creating a community of scientists working on the impacts of low pH on important micro-organisms, enabling the sharing of new concepts and methods that are being developed, but are not crossing boundaries between different disciplines and sectors’. The response of micro-organisms to low pH illustrates the points made above very well. Different micro-organisms have different abilities to survive or grow at low pH, and many encode inducible systems that extend this range. Acidity has profound effects on cell physiology, both because cells use proton gradients to power much of their important machinery, and because low pH is damaging to many components of the cell. From the applied perspective, low pH (often in the form of weak organic acids, that have multiple pH-dependent effects on cell) has been used as a preservative for food and drink since prehistoric times, as well as being a component of some traditional antibacterial treatments. Resistance to low pH is also a crucial phenotypic trait in bioprocesses that have to be conducted under acidic conditions, and in bioprocesses for overproducing the acids themselves. Such processes are important given the prominent role that micro-organisms are expected to play in the transition to a green bioeconomy. The study of acid resistance and acid stress responses thus straddles several different domains of pure and applied research, a feature it shares with many other stress responses (Lund et al. 2020, Azizi et al. 2022). But the study of acid stress is inherently a very complex one: the term ‘low pH’ covers a very wide range of proton concentrations, and the response of an organism to any one of these depends on multiple other factors, both biotic and abiotic. For this reason, responses to low pH are much more diverse in nature than those to more tightly constrained stresses such as heat shock, where very similar proteins with similar properties are induced in all organisms, from psychrophiles to thermophiles.

## Structure of the review

In this review we consider work on bacteria, yeasts, and filamentous fungi. We have not tried to summarize what is known about the nature and mechanisms of acid resistance and tolerance, and acid stress responses in micro-organisms, since these has been well-covered in other reviews on prokaryotes (Slonczewski et al. 2009, Krulwich et al. 2011, Lund et al. 2014, Guan and Liu 2020, Schwarz et al. 2022) and eukaryotes (Mira et al. 2010b, Palma et al. 2018, Guaragnella and Bettiga 2021). Instead, in this review we focus on the methods used to study different aspects of acid stress, and the experimental rationale for their use. We have chosen this approach for three reasons. First, it illustrates the wide range of approaches that have been used in the field, and shows some of the difficulties and pitfalls in designing and interpreting even apparently simple experiments. Second, we hope our approach will encourage researchers interested in acid stress to look beyond their own areas of interest and expertise and see what insights other methods may bring to their work, and use this to develop new skills and build wider collaborative networks. And third, although the review uses acid stress as the exemplar throughout, the methods presented are of relevance to studies of other stress responses and microbial growth in other potentially stressful environments, and thus should be of interest to a very broad range of readers.

**Figure 1.**
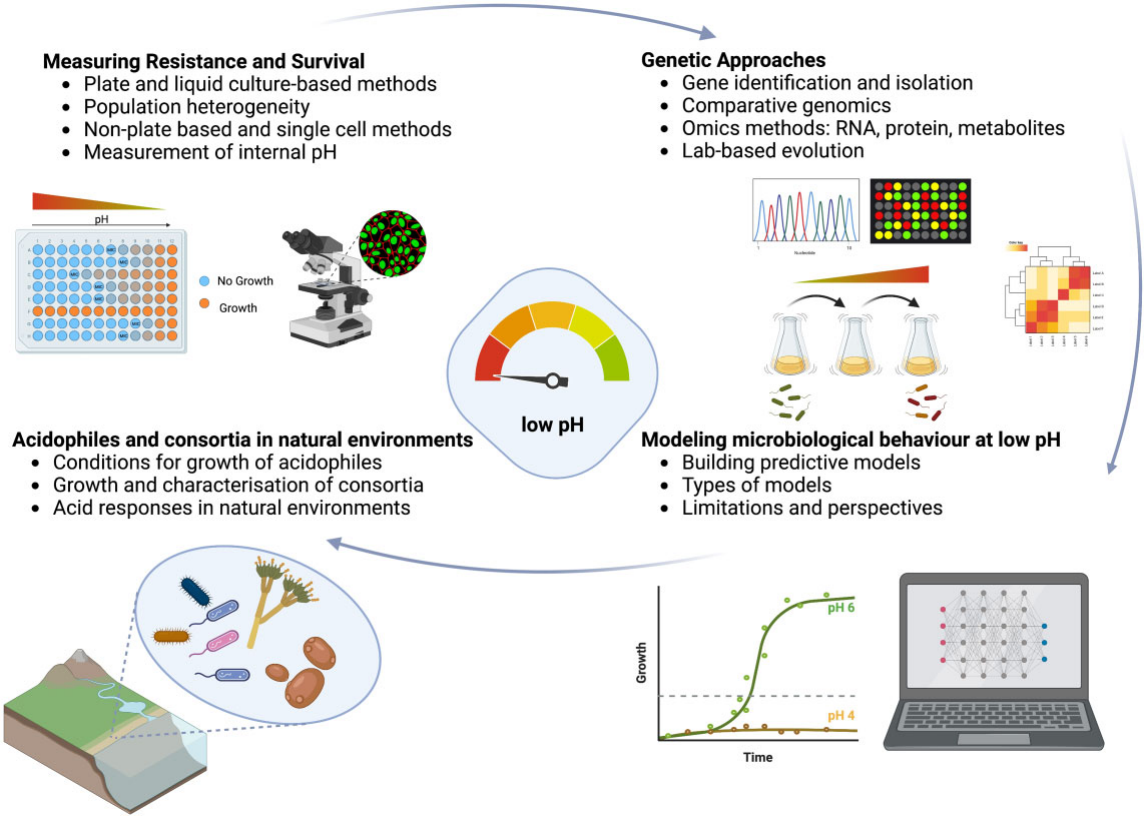
Summary of methods that are covered in this review. Figure created with biorender.com.

In this review, as shown in Fig. 1, we first set out the methods that are used to study the responses of micro-organisms to low pH where these are being considered as a laboratory-based phenomenon: that is, something that can be studied with high precision under carefully controlled conditions. These include plate-based and culture-based methods that look mostly at populations, as well as methods for individual cells such as microscopy, microfluidics, and cytometry. We consider the important issue of how to measure intracellular pH at both the whole population and the single cell level. We discuss the many approaches that are available for identifying relevant genes, their products, and the consequences of their expression, particularly in the postgenomics era. We then move our focus from studies that are mostly done on pure cultures under laboratory conditions to consider issues in more complex scenarios that are more representative of acid stress in natural environments. We begin with a discussion of the special case of extreme acidophiles, and then consider methods for the study of acid stress and acid resistance and tolerance in environmental samples, including in microbial consortia. Finally, we review some of the modelling methods that can be used to understand and predict microbial responses in complex heterogeneous environments such as foods.

## Definitions

The literature on acid stress contains many different usages, terms, and definitions, which is a potential source of confusion.

Words or phrases such as ‘acidic’ and ‘low pH’ are used in different publications to cover roughly six orders of magnitude of proton concentration, starting at just below neutrality. Reduction in pH can be caused by many different types of acids, from weak to strong. The effects of these can be distinct even at the same pH, depending as they do on properties of acids including their chemical structure, their pKa, (a measure of the extent to which they ionize at a given pH), and the nature of the anion produced. All these variables should be carefully considered in experimental design, and reported in full in publications.

The innate ability of some micro-organisms to survive at a potentially lethal low pH is generally referred to as ‘acid resistance’. This term can also be used to refer to an inducible phenotype, requiring the expression of genes that are often induced by exposure to a nonlethal pH, sometimes referred to as an ‘acid shock’. The term ‘acid tolerance’ is also used, usually to refer to an ability to grow (as opposed to merely survive) at a defined low pH. Both terms are essentially relative, involving for example the comparison of two or more species grown under the same conditions, or one species grown under different conditions, or a wild-type and a mutant strain of the same species grown under the same conditions. Any usage of these terms should, therefore, ideally state the specific acid used, the specific pH being studied, whether cells are growing or simply surviving, and the strain or condition with which the comparison is being made. It can be potentially misleading to describe cells simply as ‘acid resistant’ or ‘acid tolerant’, or indeed ‘acid sensitive’, as levels of resistance and tolerance are strongly affected by multiple growth parameters, as we will illustrate. Moreover, as we also discuss further below, distinguishing between growth, survival, and death is not always trivial.

The molecular mechanisms that lead to resistance of some micro-organisms to a normally lethal pH following prior exposure to a relatively mild acidic pH has been referred to in the literature as the ‘acid tolerance response’, ‘acid shock response’, or the ‘acid resistance response’ though again these have sometimes been used to refer to different responses shown by the same organism to different conditions. Terms such as ‘acid habituation’ and ‘acid adaptation’ that have also been used are another source of potential confusion, as development of resistance to acid may be short term, mediated by induction of a response to a moderate but nonlethal drop in pH, or longer term, caused by mutation and natural selection of more resistant strains.

In this review, we use the simple definition of acid resistance being the ability to survive a normally lethal pH, and acid tolerance being the ability to grow at a nonlethal but acidic pH. For more complicated scenarios (including the phenotypic heterogeneity present in many populations), we always explain the context in which this has been studied. We use the term acid stress response in general to refer to any microbiological behaviour that occurs as a result of exposure to a pH lower than its optimal pH for growth, and again we have always aimed to provide specific details when we refer to examples of this. Because of the many possibilities for confusion in this area, and because of some lack of clarity in the literature, we return to this issue in the final section of this review, where we suggest some minimum standards for reporting research in this field.

## Studying acid resistance and tolerance as laboratory-based phenomena

### Measuring acid resistance and tolerance

#### Methods based on plate counts and growth rates

Measuring the extent to which organisms can survive exposure to a potentially lethal pH frequently involves culturing methods to determine the number of survivors within a population. To determine the proportion of a population of microorganisms that survives a potentially lethal treatment, the simplest and most widely used method is ‘viable cell counting’ or ‘plate counting’. In this method, aliquots of the acid-treated culture are typically spread onto an agar-based medium and the colonies are counted after a suitable incubation period. This method gives an estimate of population size that is correctly expressed as colony-forming units (CFU) per ml. Since microbial population numbers are often high, the cultures are usually decimally diluted prior to plating on the agar-based medium. Multiple small aliquots (typically 5–8 μ) of 10-fold dilution series can also be spotted onto agar plates, which although less accurate than estimating numbers from individual dilutions, is quicker, uses fewer plates, and enables quick comparisons of multiple strains or treatments on the same plate.

These plating methods, though widely used, make some assumptions that are not always valid. Even defining a simple term such as ‘survivors’ can be difficult. Plating methods assume that all living cells within the population are capable of forming a colony within the timeframe of the incubation. This is not always true as some cells may be alive but injured and exhibit an extended lag phase, or even a complete failure to grow without a period of recovery, which means they go undetected within the incubation period allowed. An increase in colonies over time may thus represent recovery of viability, or actual cell division, or a mixture of the two. This problem can be partially overcome by simply extending the incubation period or in some cases by including ‘recovery’ components in the medium, such as sodium pyruvate or catalase that reduce damage from oxidative stress and help injured cells to recover (Oliver 2010, Ducret et al. 2014). Viable cell counting also assumes that each colony arises from only a single cell within the population. However, this is often not the case. If cells within the population exhibit a filamentous, clumping, or flocculating phenotype, something that may itself be a consequence of the low pH stress or part of an acid resistance mechanism (Cheng et al. 2017), then a single colony may arise from multiple cells rather than just one. In this scenario the plate count (CFU/ml) would underestimate the number of live cells (Jennison and Wadsworth 1940, Stackhouse et al. 2012, Vail et al. 2012). Notwithstanding these limitations, viable cell counting continues to be an extremely popular, accurate, and useful means of assessing changes in viability within a population that occur following treatments with a defined stress.

There are many technical factors that should be considered when using viable cell counting to measure acid resistance. If the population is to be decimally diluted prior to plating, the diluent should be buffered to an optimal pH for growth so that it does not exacerbate the lethality of the acid treatment. It should also be iso-osmotic with the medium in which the treated population is suspended, so that cells do not experience an osmotic shock, which can increase killing through activation of mechanosensitive channels and membrane permeation (Hoffmann et al. 2008, Blount and Iscla 2020). The nutrient composition of the culture medium, the diluent, and the agar medium used for plate counting can also have a significant effect on the outcome of the experiment (McFeters et al. 1982, Mian et al. 1997) so these should be chosen carefully, and clearly recorded in the description of the method, to maximize recovery; this will also enable other researchers to precisely reproduce the conditions used. It is unsurprising that a complex phenotype such as acid resistance, which is underpinned by a multitude of distinct and overlapping protective mechanisms (Foster 2004, Arcari et al. 2020, Lund et al. 2020), will be influenced by the nutrients available to it, so these need to be carefully controlled insofar as is possible. The same thing is true of the acid stress response. A good example is the inducible acid stress response of *Escherichia coli*, of which the major component is referred to as the AR2, or GAD, system. The AR2 system requires the presence of either Na^+^ or K^+^ in the medium, is strongly inhibited by indole that can itself be produced by *E. coli* from tryptophan, and is not induced at all in the most commonly used growth medium, lysogeny broth (commonly referred to as LB; Eguchi and Utsumi 2014, Boon et al. 2020). When performing experiments looking at acid resistance and acid stress responses on a previously untested strain it is therefore strongly recommended that preliminary testing is done with a range of different growth and stress conditions, to ensure that optimal cell recovery is achieved, and that stresses, other than the one under study that is caused by the low pH, are avoided as much as possible.

One important consideration when attempting to assess the acid resistance of a population is the physiological state of the cells within it at the time of measurement. It is well known for example that for populations where the rate of death is the same as the rate of cell division (e.g. cells in stationary phase), the level of resistance to low pH is generally much higher than in exponentially growing populations (Lee et al. 1994, Davis et al. 1996, Waterman and Small 1996, Lorca and de Valdez 2001). As an example, if acid resistance is followed during growth in a batch culture of *Listeria monocytogenes* it changes continually throughout, reaching a maximum in stationary phase and a minimum near mid-exponential phase (Davis et al. 1996). A similar trend is observed for batch cultures of *E. coli* (Jordan et al. 1999), and *Saccharomyces cerevisiae* (Cabral et al. 2003). Thus, when making comparisons of acid resistance between strains, the stage in the growth cycle needs to be determined, and clearly stated in any reporting of results. In some bacteria the basis for these growth-related differences is known to partly depend on stress-inducible sigma factors that become activated in stationary phase. In *E. coli, Shigella*, and *Salmonella*, the sigma factor σ^S^ (RpoS) accumulates in stationary phase leading to the transcription of a large regulon of genes that contribute to stress resistance, including several acid resistance mechanisms (Small et al. 1994, Soo Lee et al. 1995, Waterman and Small 1996). In *L. monocytogenes*, the activity of σ^B^ (SigB) increases in response to stress and stationary phase (Utratna and O’Byrne 2014) and it plays a significant role in regulating acid resistance (Wiedmann et al. 1998, Abram et al. 2008, Guerreiro et al. 2022). Related to these growth phase-dependent changes in acid resistance is the widely observed increase in acid resistance that occurs in biofilms, a phenomenon observed in many different species including *Streptococcus mutans, E. coli*, and *Lactiplantibacillus* (formerly *Lactobacillus*) *plantarum* (McNeill and Hamilton 2003, Lee et al. 2007, Kubota et al. 2009).

In contrast to measurement of acid resistance based on the ability to survive a potentially lethal acid challenge, the behaviour of cells that exhibit a degree of acid tolerance to a sublethal acid stress can be examined by recording growth rates in a specific culture medium at a range of different pH values. In this set-up, individual strains or species can be classified as more or less acid tolerant depending on the growth rates they exhibit. Since growth rates can be quantified comparatively easily by recording optical density changes over time it is straightforward to compare the acid tolerance of two or more strains (under a specific set of culture conditions). Indeed, a 96-well microtitre setup can be used to scale these comparisons up for multiple strains and multiple acidic conditions (Nygård et al. 2014, Bushell et al. 2019, Mukherjee et al. 2021), although growth in microtitre plates may be very different to that in shake flasks, due to factors such as the degree of aeration. Such comparisons can be important for modelling the growth of spoilage or pathogenic microorganisms in food applications (Koutsoumanis et al. 2016), but also have use in fundamental research on acid resistance and tolerance mechanisms (Ma et al. 2020b, Mukherjee et al. 2021). Bioreactors (that may vary in scale from a few ml to thousands of litres) often incorporate systems for continuous monitoring of growth and pH, and indeed an understanding of how production strains behave under industrial-scale conditions is often an important component of optimizing biotechnological production processes, though this is beyond the scope of the current review. However, we note that many small-scale systems are being developed for automatically monitoring microbial growth, and the parameters affecting it, and anticipate that these may become more widely used in the field of acid stress response research in the future.

A very important consideration in all growth and survival studies is that micro-organisms will often change the pH of the media as they grow. LB medium is one of the most widely used media in microbiology, but not everyone appreciates that growth of many bacteria such as *E. coli* causes LB to become more alkaline over time (due to utilization of oligopeptides in the medium as a carbon source, followed by subsequent secretion of ammonium; Sezonov et al. 2007). However, if glucose is added to LB as an alternative carbon source, mixed acid fermentation will lead instead to acidification of the medium during growth. It is therefore essential in experiments looking at the effects of altered pH that the pH is actively monitored throughout the experiment. If a specific pH is to be maintained, suitable buffers may need to be added. The best buffers to use are those that are as biologically inert as possible and have a pKa that is close to the pH range under study; the necessary concentrations to add for effective buffering need to be determined empirically (Good et al. 1966, Pielak 2021). Buffers that are commonly used in the laboratory may be worse than useless in this regard; TRIS for example is not biologically inert and its effective buffering range is pH 7–9. Most of the commonly used Good’s buffers are also ineffective much below pH 6.5. Two exceptions are 2-(*N*-morpholino)ethanesulfonic acid with a pKa of 6.15 and homopiperazine-1,4-bis(2-ethanesulfonic acid with a pKa of 4.4, both of which have been used in experiments looking at the effects of low pH on growth in bacteria and fungi (Kim et al. 2007, He et al. 2017, Bushell et al. 2020). There are many potential buffers available that have a lower pKa and/or a wider buffering range, but care has to be taken in using these in growth media since many are biologically active, either because they can be metabolized by cells or because they are organic acids which themselves are often potent antimicrobial agents. An example is McIlvaine’s buffer, which consists of a mixture of citrate and disodium hydrogen phosphate: by changing the ratios of these, the pH can be altered over a very wide range. Another is 3,3-dimethyl glutaric acid, which has been used in experiments with fungi over a range of 3–7, and was shown to be metabolically inert and to have no inhibitory effects at the concentration used (Slayman and Slayman 1970).

Growth rates are most often assessed using pure cultures, often in rich media, and it must always be remembered that these may not represent a state that is commonly met in natural environments, where competition for limited resources between multiple species is normal (Hibbing et al. 2010). Differences in acid resistance and tolerance phenotype and growth rate between different strains or between mutants in the same strain are sometimes best revealed by competition assays, where two strains are grown together and changes in their relative numbers are assessed. This method can be used to derive a direct measurement of the relative fitness of the two strains (Wiser and Lenski 2015).

In the particular case of filamentous fungi, tolerance or resistance to low pH is often evaluated respectively in terms of filamentous growth rate (measuring the radius or the area of growth on solid medium, evaluating biomass by dry weight in liquid media, or indirectly measuring biomass accumulation by optical density), or as conidial germination, monitored as CFUs by diluting conidial suspension and evaluating colonies as described above for bacteria and yeast. Also as above, CFUs do not always represent individual spores as many fungal spores are formed and remain as clumps. Filamentous growth rate is also not an ideal parameter to measure as environmental conditions may significantly affect hyphal morphology, change branching rates, and inhibit polar growth, while biomass may not be directly linked or correlated to these parameters. pH can also affect fungal morphogenesis and development, and as such is a major component of their pathogenicity, affecting traits like hyphal morphology, the development of specialized organs such as fruiting bodies, conidiophors, conidiation, sclerotia formation, appressoria, and so on (reviewed by Fernandes et al. 2017, Li et al. 2022). These are mostly evaluated by visual inspection and light microscopy, which we consider further below.

#### Heterogeneity in populations

A generalized concept of acid resistance and tolerance for a specific strain of microbe is made even more difficult to implement because microbial populations, even those that are clonal, have intrinsic heterogeneity (Ryall et al. 2012). As well as stochastic variation within the population, which arises naturally as a result of cell-to-cell differences in macromolecule and metabolite concentrations, there can also be genetic variation (through spontaneous mutation), physiological heterogeneity that occurs because cell cycles are not synchronous, and other regulatory differences discussed further below. This heterogeneity within the population means that cells will not all be equally susceptible to killing caused by low pH. The fact that subpopulations can survive for longer periods than the bulk of cells in a culture following acid treatment is evidenced by shape of the ‘kill curves’ that are generated following a typical viable cell counting experiment. A pronounced tail of survivors in this curve can indicate the presence of an intrinsically resistant population, as seen when some strains of *E. coli* cells are treated with strong acid (Benjamin and Datta 1995, Jordan et al. 1999).

Heterogeneity within clonal microbial populations can also arise due to strategies of bet-hedging that allow subsets of the population to stop growing and therefore become more resistant to environmental stresses, or to express genes that make them more resistant. In general, there is an inverse relationship between rate of cell division and sensitivity to stress, a phenomenon that arises because of a fundamental trade-off between growth and survival. When most of the resources are diverted into growth-related functions less is available for protection and repair, and vice versa (Abram et al. 2008, Phan and Ferenci 2013, Patange et al. 2018, Biselli et al. 2020). Thus, activation of a nongrowing phenotype may take place as a protective response to environmental stress conditions. The literature on slow growing or dormant microbes describes three main subtypes: viable but nonculturable (VBNC), hibernation, and persisters. Knowledge of these is important for designing experimental strategies that will genuinely detect survivors of acid stress.

The term ‘viable but nonculturable (VBNC)’ was first coined in 1982 to describe bacterial cells that were apparently metabolically active but not capable of forming colonies on standard laboratory media (Xu et al. 1982). The concept has promoted much discussion and debate over the years as to whether the VBNC phenomenon represent is a deliberate and regulated process or merely a consequence of the onset of cell death (McDougald et al. 1998, Colwell 2000, Nyström 2003). It is certainly the case that microbial cells that are physically intact and have metabolic activity (usually determined microscopically) can be incapable of forming colonies on medium that normally supports their growth. This phenomenon has been reported for over 100 bacterial species (Oliver 2010) and also for many yeast species (Salma et al. 2013, Capozzi et al. 2016, He et al. 2022), and can easily confound an experiment designed to measure survival following acid treatment. Indeed, measurements of resistance to weak acid treatment in *L. monocytogenes* have revealed that a significant proportion of the population is viable as determined by microscopic staining but incapable of forming colonies on brain–heart infusion agar (Cunningham et al. 2009). Similarly, citric acid has been reported to induce a VBNC state in *Staphylococcus aureus* (Bai et al. 2019a). The acid resistance of VBNC populations of *Vibrio vulnificus* and *V. parahaemolyticus* is known to be elevated relative to growing cells (Wong and Wang 2004, Nowakowska and Oliver 2013). Thus, to fully understand how a population is responding to an acid challenge, researchers should consider monitoring viability using a plating-independent method (e.g. fluorescent microscopy using a ‘live/dead’ stain; see below) in parallel with plate count assays.

Hibernation describes a state where under starvation conditions many bacterial species promote the production of 100S translationally inactive ribosomal 70S dimers. These hibernating ribosomes serve to reduce the capacity for protein synthesis and prevent the necessity for *de novo* ribosome synthesis when conditions favourable to growth resume (Prossliner et al. 2018, Khaova et al. 2022). The formation of these hibernating ribosomal dimers is promoted by so-called hibernation factors (HF) and their expression is typically induced in inverse proportion to the growth rate (Wada 1998). Deletion of the genes encoding the HFs produces stress sensitive phenotypes in a variety of bacterial species. For example, it is known that *E. coli rmf* mutants lacking the capacity for 100S ribosome formation have decreased acid resistance (El-Sharoud and Niven 2007).

Finally, ‘persister cells’ is a term more commonly applied to microbial subpopulations that are tolerant to antibiotics, something that can be especially problematic in treating chronic bacterial infections. They are detected primarily as a tail of highly tolerant survivors following the antibiotic treatment of a population (Lewis 2010, Kaldalu et al. 2020). In both *E. coli* and *Salmonella*, toxin–antitoxin (TA) systems have been implicated in the development of a persister cell phenotype (Hong et al. 2012, Helaine et al. 2014). In *S. aureus*, however, TA systems do not appear to play role in persister cell development. Instead, cellular ATP depletion associated with stochastic variation in the tricarboxylic acid cycle seems to be a key factor in generating this phenotype (Zalis et al. 2019). In at least some species, persister cell formation is associated with increased acid stress resistance. For example, in the acetic acid bacterium *Acetobacter pasteurianus* there is a strong link between persister cell formation and acid resistance (Xia et al. 2021). Interestingly, heterogeneity in cytoplasmic pH homeostasis has recently been reported as a contributing factor to persister cell formation in *E. coli*, with reduced pH being associated with increased resistance to antibiotic treatment (Goode et al. 2021). Indeed, *E. coli* mutants with a reduced capacity for glutamate-based pH homeostasis actually show an increased proportion of persister cells within the population (Hong et al. 2012).

#### Imaging of cells

**Figure 2.**
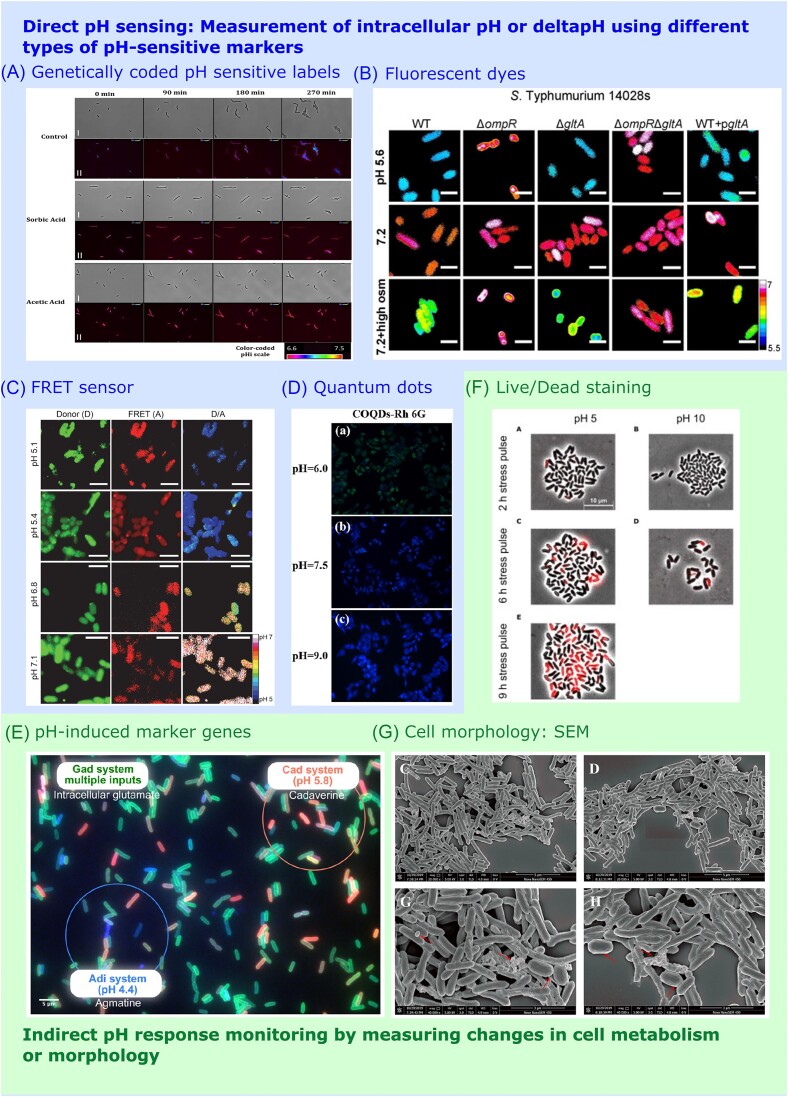
Methods for analysis of acid stress responses at single cell level. Direct pH sensing can be achieved via (A) genetically encoded pH sensitive dyes, in this case pHluorin (Pandey et al. 2016), (B) extracellularly introduced fluorescent dyes (Chakraborty and Kenney 2018), (C) FRET sensors (Chakraborty et al. 2015), or (D) Quantum dots (Wang et al. 2022). Indirect monitoring of cellular response to pH changes can be done by (E) pH-induced marker genes (Brameyer et al. 2022), (F) live/dead staining (Täuber et al. 2021), or (G) SEM (Zhao et al. 2021). Figures reproduced from cited references with permission from the publishers.

While plate-based methods are still widely and effectively used to measure the effects of low pH on microbial survival and resistance, there are many other methods for studying microbial responses to acid stress, in particular those that rely on detecting properties of individual cells, which we collectively refer to here as ‘imaging’. Use of these methods has sometimes revealed the discrepancies referred to above, when plate-based methods give a low estimate of true cell viability. Many of these methods, as summarized in Fig. 2(A), allow single-cell analyses to be performed and as such enable the examination of individuals within a population. One particularly important aspect of imaging in this field is the measurement of the internal pH of cells, which is dealt with in a separate section below.

Standard light microscopy (generally using phase contrast to visualize cells) can show effects caused by stress such as clumping or filamentation. Time-lapse real-time microscopy and quantitative image analysis are also used, for example with filamentous fungi to determine conidial germination, hyphal elongation rate, and morphology in response to various inhibitors and stressors (e.g. Aunsbjerget et al. 2015). Alternatively, scanning or transmission electron microscopy give high resolution information on changes in cell appearance and ultrastructure, although the fixation methods required can produce artefacts. SEM is mostly used for investigating surface texture and overall cell appearance of cells. Normal live bacterial cells usually have a plump appearance with a smooth surface, but acidic growth conditions can in some cases induce shrinkage, irregular shape and elongation (Zhao et al. 2021, Li et al. 2023; see examples in Fig. 2G). Force-field atomic microscopy has been used together with the above methods to investigate aspects of the morphology of acid-stressed yeast cells, revealing effects such as changes in intracellular volume, increased stiffness of the cell wall and increased tendency to flocculation (Goossens et al. 2015, Ribeiro et al. 2021). Fluorescence microscopy is widely used in the acid stress field, where the signal from fluorescent probes is measured to assess different properties of the cell that collectively can be used to infer the viability or other properties of individual cells (Breeuwer and Abee 2000). For example, membrane integrity is a key determinant of cell viability, so cells that can exclude nonpermeable fluorescent dyes are considered to have intact cell membranes. Classically propidium iodide (PI), a nonpermeable, fluorescent, nucleic acid-intercalating dye, is used to determine if cell membranes are intact. Cells that stain with PI are considered to have lost membrane integrity and are hence considered to be nonviable (e.g. Bunthof et al. 1999, Berney et al. 2007, Täuber et al. 2021; see Fig. 2F). Fluorescein-based probes can also be used to assess membrane integrity on the assumption that fluorescein can only accumulate intracellularly when the cell envelope is intact. It is typically conjugated to other chemical groups (e.g. fluorescein diacetate, FDA or carboxyfluorescein, CF), which must first be cleaved by intracellular esterases to liberate the fluorescent moiety. Tetrazolium-based dyes can be used to identify cells and fungal hyphae that are metabolically active, since these dyes are reduced to the fluorescent species formazan by dehydrogenases within the cytoplasm of viable cells (Brul et al. 1997, Kumar and Ghosh 2019). For example, the dye 5-cyano- 2,3-ditolyl tetrazolium chloride rapidly turns into an insoluble red fluorescent formazan product via the activity of the electron transport chain, and so can be used to measure respiratory activity. Many studies using fluorescent microscopy to assess cell viability employ the so-called LIVE/DEAD BacLight kit (supplied by Invitrogen) that includes both PI and the permeable nucleic acid-binding molecule SYTO9 that has green fluorescence (e.g. Chen et al. 2020b, Zhao et al. 2021, Li et al. 2023). This kit can also be used to determine viability of fungal spores (Chen and Séguin-Swartz 2010). Cells staining green or red are assumed to be alive or dead, respectively. All the above methods have to be used with caution and are not necessarily unambiguous in what they find: researchers often find that intermediate states exist within populations, giving rise to a sort of Schrödinger’s cell scenario where the cell is simultaneously alive and dead (Berney et al. 2007, Emerson et al. 2017; note that Emerson et al. (2017) summarize many methods for determining whether cells are viable or not, not all of which have been used in studies on the effects of low pH). Cell death is part of a dynamic process of change, and time-lapse experiments may be needed to follow cell fates more precisely. If fluorescent microscopy is coupled to time-lapse imaging the additional possibilities for following changes in physiology (or gene expression if suitable fluorescent reporters are used) in response to a stimulus are almost endless. Recently this approach has been used to follow acid-induced changes in gene expression in single *E. coli* cells. By combining *in vitro* evolution using killing caused by low pH as the selective regime with time-lapse fluorescence microscopy, the authors were able to isolate a gain-of function mutation in the sensor kinase EvgS, a known regulator of acid resistance in *E. coli* (Van Riet et al. 2022).

**Figure 3.**
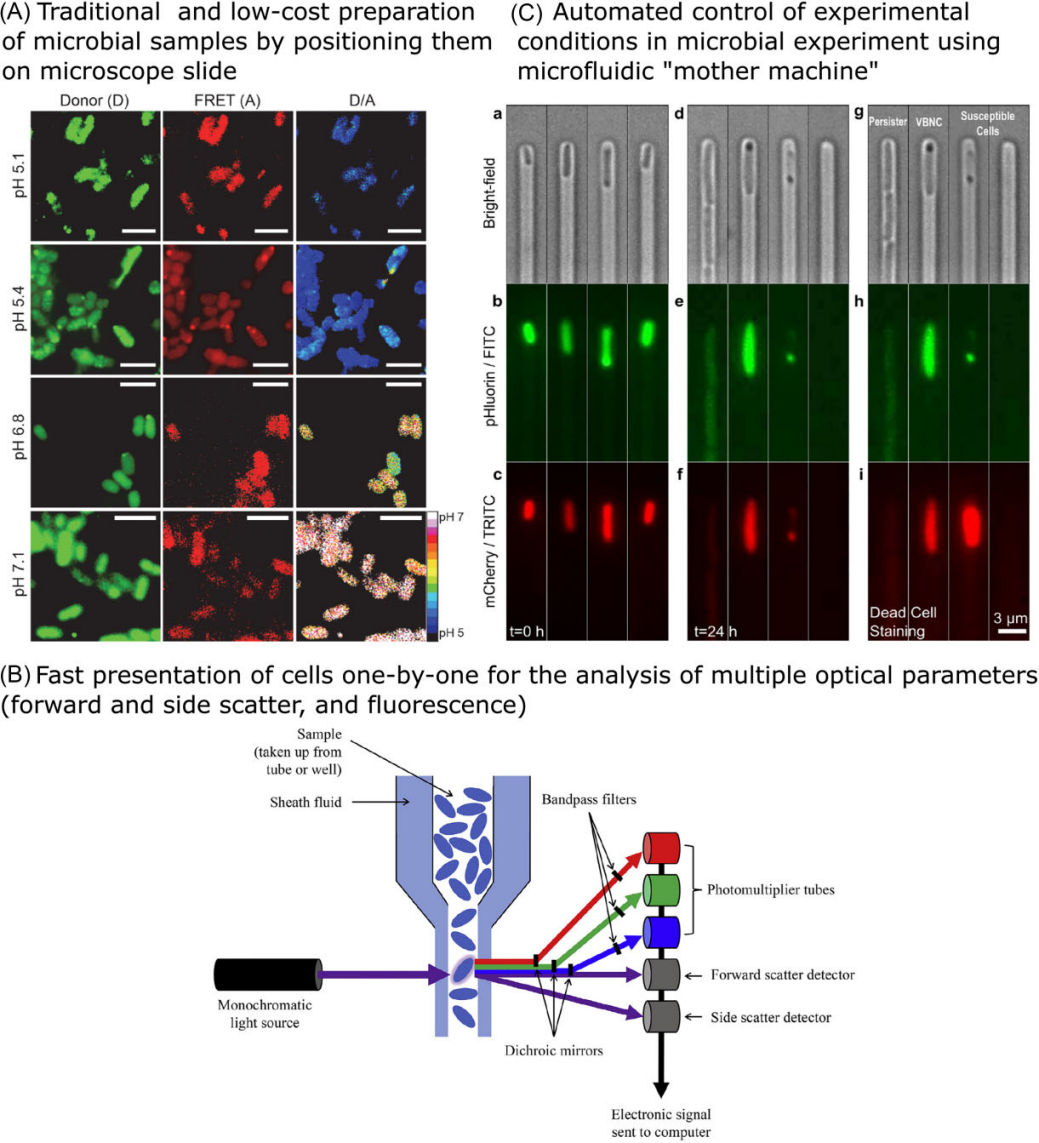
Positioning of cells for analysis with specific sensing approaches The positioning of the sample usually falls under one of the three main types: (A) traditional microscopy slide (Chakraborty et al. 2015), (B) fast single-cell analysis by cytometry (Safford and Bischel 2019), or (C) microfluidic devices (Goode et al. 2021). Figures reproduced from cited references with permission from the publishers.

For microscopic single-cell analysis of pH stress responses (and measurement of internal pH in single cells, discussed in detail in the next section), positioning of the analysed bacterial cells for detection is as important as the sensing method itself. Many positioning options exist, from widely used standard microscopy slides to different advanced microfluidic technologies. The most widely used method to position cells for single-cell analysis is via pretreated or untreated microscopy slides, (see Fig. 3A). Both live and fixed bacteria can move on a slide, due to motility and Brownian motion, respectively, making them difficult to image. One frequently used alternative is to use glass slides and variously modified cover slips. The use of cover slips made from agarose enabled Narayana et al. (2020) to perform high resolution bright field imaging using oil-immersion light microscopy. Cell size could clearly be distinguished and was used for validation of simultaneous flow cytometry results. Pandey et al. (2016) took it one step further and used a regular cover slip with a thin agarose-medium pad to construct a closed airtight chamber for aerobic bacteria. This immobilized the bacteria and allowed undisturbed growth, creating optimal imaging conditions for time-lapse fluorescence microscopy. An example of a commercially available modification is a gold antifade reagent that is sealed with entellan that can enhance long term storage of the fixed samples (Chakraborty et al. 2015).

A developing technique is the use of microfluidic devices, which can be used to trap individual cells within confined grooves, typically etched in polydimethylsiloxane, allowing for targeted manipulation and study of individual cells, something that is not typically possible within a liquid culture (Cabeen et al. 2017, Potvin-Trottier et al. 2018). The power of microfluidics comes from the fact that individual cells can be tracked over several generations allowing large data sets to be generated from populations subjected to particular stimuli. This is especially useful for studying responses to stress such as acidification, and can reveal examples of population heterogeneity in survival or growth rate. Custom-made set-ups for tracking individual cell responses can be devised, including separate culture chambers for controlled adjustment of buffers, pH, and other environmental parameters. Strains engineered to express fluorescently tagged proteins from a promoter of interest (discussed more below) can be used to learn about regulatory effects at a single cell level. This approach has recently been used to investigate the activation of the SigB-mediated general stress response in *Bacillus subtilis*. The activation of SigB in individual cells in response to a mild acidification stress was observed to be largely synchronous (unlike other stressors such as ethanol) but the response occurred transiently within the population (Hamm et al. 2022).

Different devices are now becoming available as microfluidics methods grow in popularity. One such example is CellASIC ONIX (Mitosch et al. 2017). This device allows switching between inlets containing different substances, thus allowing continuous exposure of the same cells to various conditions, which equilibrate within minutes. The device also has a preheated chamber where the bacteria cells can be spatially distributed, allowing continuous monitoring of single cells via microscopy. Another option in microfluidics is using a ‘mother machine’ device. These devices have channels where cells can grow, oriented at a 90° angle to a trench where growth medium is passed, allowing diffusion of fresh medium into the growth channels where cells are dividing and removal of older cells as they are pushed out from the channels into the trench. The device thereby makes it feasible to follow individual cells for several generations (Wang et al. 2010). Goode et al. (2021) applied time-lapse microscopy to measure bright field and fluorescence levels of cells inside the mother machine. By flowing sub-MIC levels of ampicillin, followed by flushing with growth medium, the internal cell pH response of individual cells could be monitored continuously (see Fig. 2C). The use of microfluidics has been recently also applied for single cells studies in yeast, as well as in filamentous fungi (Richter et al. 2022).

Flow cytometry is another method that offers a powerful quantitative approach to studying stress responses within microbial populations (Davey and Kell 1996, Veal et al. 2000, Müller and Nebe-von-Caron 2010, Arvaniti and Skandamis 2022; see Fig. 3).

Flow cytometers allow the detection of single cells flowing past a laser probe, through the detection of changes in light scattering and/or fluorescence. Both a total particle count and fluorescent properties can be determined. Measurements of cell sizes can be obtained by high-throughput measurement of forward light scatter (FSC) (hundreds of measurements per second). Nevertheless, it is important to use other complementary imaging or visual methods in order to confirm flow cytometry results. This is because the FSC signal is not only dependent on cell size, but also on the orientation of cells when passing through the laser beam, and on the refractive index, all of which can generate a nonlinear relationship between the FSC signal and cell size. For example, rod-shaped cells such as *Lactobacillus acidophilus* tend to align along their long axis when travelling through the laser beam, making cell size seem greater than it really is (Narayana et al. 2020). Flow cytometry has also been extensively applied on the yeast forms and conidia of filamentous fungi, and microencapsulation may facilitate its use to study hyphae or microcolonies of filamentous fungi (Bleichrodt and Read 2018, Steiger 2021).

Fluorescent probes (either added as dyes or expressed internally as fluorescent proteins; see Table 1 for some examples) can provide information on, among other things, the physiological states of individual cells, including information on structure, membrane integrity, nucleic acid content, enzyme activity, and protein expression, (Davey and Kell 1996, Veal et al. 2000, Müller and Nebe-von-Caron 2010). With the correct choice of fluorescent probes, flow cytometry can also be used to monitor cytoplasmic pH distributions within populations of bacteria or yeast (Graber et al. 1986, Breeuwer et al. 1996, Valli et al. 2005). When cell sorting is added to flow cytometry (known as fluorescence-activated cell sorting or FACS) the result is a powerful instrument capable of sorting cells based on properties defined by the user. In one study aimed at identifying acid-induced genes in *Salmonella* a variant of this approach called differential fluorescence induction was developed. A library of *Salmonella* promoter sequences was coupled to a gene for green fluorescent protein (GFP), and the resulting mixture was screened by FACS for cells that were fluorescent at pH 4.5 but nonfluorescent at pH 7.0. By combining this screen with cell sorting, individual fusions could be fished out of the library with the desired profile of gene expression, leading to the identification of several novel acid-induced target genes (Valdivia and Falkow 1996).

**Table 1.** Examples of probes that can be used for determination of intracellular pH.

Probe/method	Uses and limitation	References
Radiolabelled weak organic acids (e.g. ^14^C benzoic acid or acetylsalicylic acid)	Radiolabelled weak acids that permeate the membrane in the undissociated form accumulate intracellularly in a manner that depends on pHi. Cell volume must be determined to calculate the pH_i_. Cannot be used in single cells or for transient events.	Krebs et al. (1983)
^31^P NMR methylphosphonate (MeP)	Displays a pH-sensitive chemical shift that can be detected by NMR. pKa 7.74 and 2.38. Requires an NMR instrument and expertise for use. Cannot be used in single cells or for transient events.	Slonczewski et al. (1981)
5(6)-carboxy-fluorescein diacetate succinimidyl ester (CFDA-SE) and derivatives	Ratio of fluorescence at 490 nm (pH dependent) to 440 nm (pH independent) allows pH_i_ to be determined in individual cells thus providing information about population heterogeneity. pKa 6.5. Efflux pumps may remove probes, but cytoplasmic esterases facilitate their retention promoting intermolecular cross-linking	Breeuwer et al. (1996)
2′,7′-bis-(2-carboxyethyl)-5-(and 6-) carboxyfluorescein acetoxymethyl ester (BCECF-AM)	Ratio of fluorescence at 500 nm (pH dependent) to 440 nm (pH independent) allows pH_i_ to be determined ratiometrically. The ester is cleaved intracellularly by esterases allowing the BCECF dye to be retained. pKa 6.97. Mixture of three different isomers, relative levels of which may vary between batches	Magill et al. (1994)
I-Switch	A tetrameric DNA-based probe whose structure changes in a pH-dependent manner producing a fluorescence resonance energy transfer (FRET) signal. Requires electroporation into cells.	Modi et al. (2009)
Seminaphthorhodafluor-4F 5-(and-6) carboxylic acid (C-SNARF-4)	A fluorescent ratiometric probe that allows pH quantification independent of probe concentration and/or laser intensity. Useful for extracellular pH determination in biofilm microenvironments. pKa 6.4.	Hunter and Beveridge (2005)
pHrodo Green AM	Fluorogenic probes that traverse the cell membrane and remain within the intracellular space upon cleavage by nonspecific esterases (useful pH range 4.0–9.0). pKa 6.5	Dulebohn et al. (2017)
GFPmut3	Highly fluorescent variant of GFP with pH-sensitive emission; intensity based so reading are affected by levels of expression.	Cormack et al. (1996)
pHluorin and derivatives	pH-sensitive ratiometric pHluorin is a GFP variant that displays a bimodal excitation spectrum with peaks at 395 and 475 nm and an emission maximum at 509 nm; can be used for ratiometric sensing. Low signal of pHlourin is improved by using pHluorin 2. Use of a superfolder variant (sfpHluorin) eanbles use in more oxidizing environments (e.g. *S. cerevisiae* ER). Excitation light wavelength may have specific effects on some bacteria that are sensitive to blue light.	Miesenböck et al. (1998), Mahon (2011), Reifenrath and Boles (2018)
pHaROS	Genetically encoded biosensor for simultaneous real-time detection of changes in both redox potential and intracelular pH	Zhao et al. (2019)
pHlash	Genetically encodable and ratiometic pH probe that utilizes bioluminescence resonance energy transfer (BRET) to *in vivo* measure intracellular pH.	Zhang et al. (2012)

### Direct measurement of internal pH

Measurements of internal pH (pH_i_) are particularly valuable in studies on responses to low pH. Ideally, such measurements should be usable over a short time frame so as to reveal the kinetics of internal pH change in response to external conditions. In addition, methods that enable determination of pH_i_ in individual cells are valuable, because, as discussed above, measurements taken on whole populations are averages from large numbers of cells and can miss biologically important cell-to-cell variation.

In early investigations in this area, most work focussed on the measurement of △pH (the difference between internal and external pH) as a component of the proton motive force. This often had to be measured under equilibrium conditions (so gave no information on the rate at which pH_i_ changed in response to acid stress) and could only be done on relatively large numbers of cells. These methods involved measuring the equilibrium distribution of chemical species that show pH-dependent ionization, such as weak organic acids, between the insides and outsides of cells. Application of these methods established that although pH_i_ varies between individual species, it tends to be fairly constant over a range of external pH values. In other words, many micro-organisms display cytoplasmic pH homeostasis, which is often a key component of their ability to survive changes in external pH. Because these were not kinetic methods and could only be done on large numbers of cells, they will not be discussed further here; details with examples and caveats about their use can be found in several reviews (Padan et al. 1981, Booth 1985, Kashket 1985, Slonczewski et al. 2009).

More rapid methods involve the measurement of a pH-dependent parameter that can provide a direct value for pH_i_ after calibration against solutions of known pH. An early example was ^31^P, which shows a chemical shift with changing pH that can be measured using NMR (Slonczewski et al. 1981, 1982). As well as its use on bacterial cultures, this technology has been used to discriminate between cytosolic and intracellular pH in fungal systems including *S. cerevisiae* and *Aspergillus niger* (Shanks et al. 1990, Hesse et al. 2000). However, this requires large, concentrated cell volumes and expensive equipment. A better output for pH_i_ is fluorescence, which can be quantified with high sensitivity and in real time, on bulk populations but also, using fluorescence microscopy and flow cytometry, in individual cells. Fluorescence may arise from externally added dyes that are taken up by cells, or by fluorescent proteins that are expressed inside the cell, and may be measured directly or through FRET (Förster- or fluorescence–resonance energy transfer). We will discuss these in turn.

Various compounds (often derived from carboxyfluorescein) that show a pH-dependent change in their spectra (intensity or wavelength, or both), or which are produced by cellular catalysis of a nonfluorescent precursor in a reaction that is itself pH dependent, have been used to determine pHi. Use of these fluorescent probes enables measuring the dynamics of change following a change in external pH (Shechter et al. 1982, Breeuwer et al. 1996, Siegumfeldt et al. 1999). These dynamics were found in general to be quite fast (e.g. with adjustment of pH_i_ in some lactic acid bacteria (LAB) taking between 2 and 10 min, depending on the species) and to vary between species (Siegumfeldt et al. 2000). Measurement of internal pH in yeast cells using fluorescent probes were important in showing cell to cell variation, and also in revealing the high impact that weak organic acids can have on internal pH (Bracey et al. 1998, Viegas et al. 1998, Simões et al. 2006). The probes used have to show a number of properties to be useful, in addition to a suitable pH-dependent fluorescence signal. Ideally, they must not bind to any components of the cell or be actively transported by it, but instead must freely diffuse into the cell, and have no confounding effects (e.g. toxicity or osmotic pressure). Calibration against known pH values also needs to be done on the compound inside the cell. In bacteria, this is generally done by removing the pH gradient that is present across the cytoplasmic membrane (i.e. ensuring the value of △pH across the inner membrane is zero) so that the internal pH (pH_i_) and the manipulable external pH are the same. Compounds used to collapse the pH gradient include sodium benzoate, carbonyl cyanide *m*-chlorophenyl hydrazone, or nigericin, all of which effectively make the inner membrane permeable to protons, although the use of sodium benzoate has been called into question (Chakraborty et al. 2017, Kenney 2024).

Newer fluorescent probes that can enter cells and show a high pH-dependent signal such as pHrodo^™^ Green have been used in bacterial cells, for example to show that a decrease in the pH_i_ of *Borrelai burgdorferi* leads to the induction of stress responses that includes expression of several important virulence factors (Dulebohn et al. 2017). The probe BCECF-AM, another carboxyfluorescein derivative that fluoresces when cleaved by cytoplasmic esterases, has been used to measure the acidification of the *Salmonella* cytoplasm upon phagocytosis, which leads to secretion of important pathogenicity effectors (Chakraborty et al. 2015, 2017, Chakraborty and Kenney 2018, Zhao et al. 2021; see Fig. 2B). The Kenney group has also pioneered the use of the I-Switch to measure pHi. This is a DNA-based biosensor, introduced into cells by electroporation, which has FRET donor and acceptor groups at its ends. The FRET signal between these is lost at neutral pH but as the pH drops, the biosensor adopts a conformation that allows FRET to be measured. Another example of a FRET-based pH probe is a fluorescent rhodamine derivative that has been used successfully in *Candida albicans* (Li et al. 2018). A different fluorescent probe (C-SNARF-4) has been used to measure the pH_i_ of *S. cerevisiae* cells (Valli et al. 2005). This study showed that stationary phase cells were more able to maintain a stable pH_i_ as the external pH varied, and used flow cytometry to show the presence of subpopulations of cells with distinct pH_i_ values.

It is important to note the potential for artefacts when using fluorescent dyes: e.g. in *A. nidulans* some pH-sensitive probes are found not to label the cytoplasm but to accumulate preferentially in a cell compartment of variable size (von Recklinghausen et al. 1993). A further issue is that intensity measurements are strongly affected by how well dyes are taken up by cells (loading) as well as by pH, so data from dyes that only show an intensity change with pH must be interpreted with caution. For this reason, ratiometric dyes are often preferred: these show two emission peaks, one of which shows an altered intensity with pH and one of which does not. Measuring changes in the ratio of the two compensates for differences in loading. Many of these have been devised for use in eukaryotic cells to look at differences in different compartments (as recently reviewed by Munan et al. 2023), and have potential for use in microbial systems that has not yet been fully explored.

Nanomaterials called carbon quantum dots have also recently gained attention for being excellent fluorescent probes in pH_i_ analysis (Bai et al. 2019b, Wang et al. 2022). Carbon–oxygen quantum dots (COQDs) combined with rhodamine 6 G (Rh 6 G) are useful for both single-cell ratiometric pH sensing and antioxidant analysis, while being nontoxic and environmentally friendly. The colour change of COQDs-Rh 6 G probe showed linear correlation with the intracellular pH range of 5.94–9.7 in eukaryotic cells (see Fig. 2D). For bacteria, to date only the toxicity of this probe has been tested, so applicability for sensing intracellular pH response in bacteria cells needs further investigation.

An alternative to adding pH-sensitive fluorescent dyes to cultures is to use internally expressed proteins as pH sensors. Some examples of the many known are given in Table 1; for much more detailed information about the many different GFP variants, the reader is referred to the excellent web site at https://www.fpbase.org/. Currently, the most used examples are variants of GFP that have been optimized to show a pH-dependent shift in signal. These can be expressed at high levels to enable sensitive measurements, sometimes at single cell level (for a review of the many applications of variants of this protein in microbiological studies, see Cambré and Aertsen 2020). Advantages of using GFP include that it is produced inside the cell from cloned copies of the gene, and that it is cytoplasmic (unless engineered to be secreted), eliminating any background signal from external probe. Various mutants of GFP have been developed to improve their use as pH probes. In one example, pH-dependent changes in emission peak intensity of GFPmut3 (GFP S65G S72A; Cormack et al. 1996) were used to convert an intensity signal into a measure of pH_i_. Yellow fluorescent protein can be used in the same way, but it has a weaker signal. This probe was used to confirm that under the conditions studied the pH_i_ of *E. coli* was 7.6, and that after acidification of the external medium to pH 5.5, there was a rapid (within seconds) drop of pH_i_ to between 6 and 6.5, followed by an apparent two-phase recovery to pH 7.6 (Wilks and Slonczewski 2007). Interestingly, this transient loss and biphasic regain was not seen when using either the I-Switch or BCECF-AM described above. Reasons for this discrepancy have been proposed (Chakraborty et al. 2017, Terradot et al. 2021, Kenney 2024), and this finding emphasizes the need for caution when interpreting data obtained using different methods for determination of pH_i_. Use of GFPmut3 has suggested a critical link between antibiotic killing mechanisms and the control of cellular pH in *Mycobacterium smegmatis* (Bartek et al. 2016). The same protein can be modified with the attachment of a signal sequence for the TAT secretion system (that enables secretion of the fully folded protein, as GFP cannot fold *de novo* in the periplasm), enabling measurement of pH in the periplasm. This approach has been used in other bacteria, e.g. in the stomach bacterium *Helicobacter pylori*, which has a pH_i_ the same as *E. coli* and also shows a rapid drop and biphasic recovery after external acidification (Wen et al. 2018).

One criticism of this approach is that it is vulnerable to artefacts caused by different levels of expression of fluorescent proteins between cells, something that can sometimes be clearly seen using fluorescent microscopy (Chakraborty et al. 2017). To overcome this problem, Rhee et al. (2020) developed an analysis scaling approach to make fluorophores independent of their expression levels. One can use a ratiometric approach as described above for fluorescent dyes to overcome loading artefacts, and this approach is widely used with the GFP-derived protein pHluorin and its derivatives, which has excitation peaks at 395 and 475 nm (Mahon 2011, Pandey et al. 2016; see Fig. 2A). The former decreases and the latter increases as the pH drops, and by appropriate calibration the ratio of the two can be converted into a value for pH_i_ that is not affected by the level of expressed probe, and hence compensates for any cell-to-cell variation in expression levels. pHluorin variants engineered for expression in different organisms have been widely used to measure pH_i_ in bacteria including *E. coli, B. subtilis, L. monocytogenes, Pseudomonas* spp., *S. cerevisiae*, and *A. niger* (Young et al. 2010, Martinez et al. 2012, van Beilen and Brul 2013, Pandey et al. 2016, Crauwels et al. 2018, Arce-Rodriguez et al. 2019, Shin et al. 2020). Single cell studies using pHluorin probe highlight the existing cell-to-cell heterogeneity in pH_i_ caused by subpopulations that can subsequently lead to altered survival of some cells (Pandey et al. 2016, Mitosch et al. 2017). Similar findings of cell–cell heterogeneity have been revealed using ratiometric probes in yeast, and these have also demonstrated a direct link between cell division rates and cytoplasmic pH (Orij et al. 2009, Fernández-Niño et al. 2015, 2012). Another approach to removing the problem of heterogeneity of expression is to use an mCherry–pHluorin translational fusion protein, as mCherry fluorescence is not affected by pH (Goode et al. 2021) so can be used to normalize the pHluorin signal.

An unrelated drawback of the use of GFP and its derivatives is that it is excited by light in the blue/violet end of the spectrum: many bacteria (including *E. coli*) are particularly sensitive to light at these wavelengths. pH-sensitive derivatives of the fluorescent mCherry protein have been produced to avoid this issue, and they produce results consistent with the analysis of *E. coli* done using GFP derivatives; in this case, the signal measured is the lifetime of the fluorescence signal rather than its intensity, which also avoids the problem with variable expression (Haynes et al. 2019). Photobleaching can also be an issue with GFP, particularly in time-lapse experiments. The development of new more photostable variants is helping to reduce this problem (Hirano et al. 2022). In general, as with all fluorescence-based assays, the final choice of probe used will depend on experimental circumstances, including the quality of experimental equipment: measurements need to made at a level appropriate to the sensitivity and linear range of the equipment used.

In conclusion, a wide range of methods has been used to measure pH_i_ both in whole populations and in single cells of many different micro-organisms. However, to yield reliable data, these methods have to be applied carefully with proper controls and calibration, and ideally verified using independent methods. New fluorescent dyes and proteins that show pH-sensitivity are constantly being described in the literature, often for work on nonmicrobial systems, and examples of some of these are shown in Table 1. We are confident that work in the field of microbial acid stress will continue to benefit from their use.

### Genetic analysis of acid resistance and the acid stress response

#### Classic approaches

The rationale for studying the genetic basis of any biological phenomenon is based on the well-established observation that identification and analysis of the relevant genes and the products they encode can lead to a fuller understanding of the biological processes in which they are involved. Microbiology operates today in the postgenomic world, and many of the methods used in studying the genetics of acid resistance and acid stress responses are hence reliant on the production and analysis of ‘omic data of various kinds, as discussed below. However, some of the most important early findings in the field were made not only before the structure of DNA was known but even before its role as the genetic material was recognized. It was shown a century ago (Hanke and Koessler 1924) that some bacteria could decarboxylate amino-acids, and these authors insightfully proposed that this process might represent a protective mechanism used by bacteria against low pH. Subsequent work by Ernest Gale and others (summarized in Gale and Epps 1942) showed that these decarboxylases were only produced when bacteria were grown in media at a relatively low pH (typically around pH 5) that is now known to be near-optimal for induction of resistance to very low pH in *E. coli* (Burton et al. 2010). Studies on these proteins assisted the early cloning of some of the genes important in acid resistance, such as the *gadA* and *gadB* genes of *E. coli* that encode glutamate decarboxylases (De Biase et al. 1993, 1996) and the *cadA* gene of *E. coli* that encodes lysine decarboxylase. It is instructive to look at this second example in more detail, as some of the basic principles illustrated remain true even in the post-‘omics era. It also remains the case that inferences of function from genetic data are always strengthened by biochemical confirmation, ideally using purified components. These days, the biochemistry usually follows the genetics, rather than facilitating it.

The classic ‘forward genetics’ approach is to identify mutants where this phenotype of interest is altered, and then find the gene(s) that have been mutated. Two groups used this approach to identify the *E. coli cadA* gene in 1992. One built on the initial isolation of a mutant strain lacking lysine decarboxylase activity, which was found using a brute force approach of random mutagenesis following by screening of individual colonies for loss of activity (Tabor et al. 1980). A similar screening of random mutants for loss of activity identified the same gene, but in this case the mutation was made by random insertion of a transposon (Auger et al. 1989). The gene identified in these two studies was mapped using a range of microbial genetic methods (now redundant in the genomics age), and sequencing of this gene, and comparison of the predicted with the actual N-terminal amino-acid sequence derived from the purified protein, confirmed identification of the *cadA* gene (Meng and Bennett 1992). An alternative approach used random insertion of a transposon carrying a promoterless *lacZ* gene, followed by screening of individual colonies for pH-inducible *lacZ* expression. One such colony was subsequently shown to carry the transposon in the *cadA* gene (Slonczewski et al. 1987, Watson et al. 1992).

Similarly, using forward genetics, the regulation of gene expression in response to extracellular pH has been studied in filamentous fungi. Many fungi can grow at a very broad range of pH (i.e. 2–10) by adjusting their metabolism and specifically their extracellular secreted enzymes in accordance. Early work used high level irradiation of *A. nidulans* conidia with UV, followed by plating and histochemical staining of colonies *in situ* to reveal individual mutants showing an increase or decrease in acid or alkaline phosphatase activity (Dorn 1965). Many of these were later shown to be mutants of a central transcription factor, PacC, that regulates gene expression in a pH-dependent manner in many fungi and that was subsequently found to regulate many metabolic, morphogenetic, developmental, and pathogenic processes in response to environmental/host pH (reviewed in Arst and Peñalva 2003, Peñalva et al. 2008, Li et al. 2022).

Identification of genes by classical methods, combined with the development of molecular genetics as a discipline, opens the doors to numerous follow-on experiments. DNA sequencing of the regions adjacent to the gene may reveal other nearby genes with roles in the same process (often expressed in the same operon); both *cadB* (a lysine:cadaverine antiporter) and *cadC* (a transcriptional activator) were identified in this way following the isolation of *cadA*. Construction of targeted mutations in such genes can help to discern their function by looking at the phenotypes of the resulting strains. The ease of making such mutations varies in different organisms, but with the development of new tools, including CRISPR-based methods, direct engineering of genomes is becoming more universal. The availability of the cloned genes also facilitates expression and purification of their protein products, which can then be used to add biochemical weight to the genetic analysis. Gene sequences can also be used to look for homologues in other bacterial species, via BLAST searches of all sequenced genomes. This approach has been used, e.g. to map the occurrence of *gad* genes in enteric bacteria (Pennacchietti et al. 2018).

An alternative and widely used route to studying expression of individual genes, once identified, is fusion of their upstream regions to various reporter genes, usually on a plasmid. Ideally, such plasmids should have a relatively low copy number, to avoid titrating out regulator proteins that may be present at low levels, and the reporter genes need to encode proteins that are easy to analyse. Bacterial luciferase is a particularly useful reporter as its activity can be measured nondestructively in live cells in real time, enabling high-resolution dynamic studies of changes in gene expression following acidification (e.g. Burton et al. 2010). It can, however, only be used in aerobically grown cells, so cultures expressing bacterial luciferase need to be kept well aerated to yield consistent results. Also, luminescence does not provide a direct measurement of promoter activity, which requires further data processing (Iqbal et al. 2017). Site-directed mutagenesis of these upstream regions enables the various cis-acting regulatory elements in promoters to be identified. Transcriptional fusions can also be used in screens to identify unlinked regulator genes. Most work using reporters of this type has been done on whole populations, but the development of highly fluorescent reporters now enables analysis at the single cell level, often revealing high levels of heterogeneity across populations (e.g. with the *cad* system see Fritz et al. 2009, Brameyer et al. 2020). Creation of a reporter strain with separate colours for each of the main acid resistance systems (*E. coli gadC*:eGFP-*adiC*:mCerulean-*cadB*:mCherry), enabled for the first time a simultaneous quantitative investigation of the activity of each system in individual cells, which was found to be heterogeneous in nature within isogenic populations (Brameyer et al. 2022, see Fig. 2E).

#### Insights from comparative genomics

The wide availability of fully sequenced microbial genomes opens up almost endless possibilities for studying the evolution of individual genera, species, and strains in order to gain insights into their niche-specific adaptations and fundamental biology. While the vast quantity of sequence data now available presents its own challenges, especially to classically trained microbiologists who have limited first-hand experience with computational biology, the continuous improvements in computational power, software development, and artificial intelligence are presenting lots opportunities for researchers. At the simplest level, it is generally the case that once a gene has been found to play a role in acid resistance, whether through classical or postgenomic approaches, the first thing most researchers do is to use homology search tools such as BLASTP to see how widespread it is and what is already known about it. Similarly, anyone interested in possible acid resistance mechanisms in a novel organism is likely to inspect the genome sequence first, and to test possible examples by engineering deletions of candidate genes and testing their phenotype (a process referred to as ‘reverse genetics’, to distinguish it from the classical ‘forward genetics’ approach described above, where finding mutants with a phenotype of interest precedes identification of the gene). Care must be taken not to assume either that all functional annotations of gene sequences or genomes are correct, or that sequence homology necessarily means that two proteins have the same function. Going beyond this, one powerful approach that aids understanding of the genetic factors that determine phenotypes of interest is an approach collectively called genome-wide association studies (Chen and Shapiro 2015, Power et al. 2017). This approach allows the phenotypic properties from multiple microbial strains (the larger the number the better, because of the reliance on advanced statistical analysis) to be correlated with their whole genome sequences. The aim is to find genetic markers that are highly correlated with a specific trait of interest. When this approach is applied to an individual species it can give new insights into the genetic factors linked with pathogenicity (Kim et al. 2020b, Chaguza et al. 2022), antimicrobial resistance (Kavvas et al. 2018, Farhat et al. 2019, Ma et al. 2020a), and stress tolerance (Neshich et al. 2013, Yahara et al. 2017, Fritsch et al. 2019). An example of such an analysis of the frequency of premature stop codons occurring in the 22 340 publicly available *L. monocytogenes* whole genome sequences showed there were unexpectedly high rates in genes of the *sigB* operon that act positively on SigB function (Guerreiro et al. 2020). These findings suggested that this pathogen must encounter evolutionary pressures acting to select for SigB loss of function, with a negative consequence for acid resistance. Other methods can be used for comparative studies using smaller numbers of genomes. Recently, Wu et al. (2022, 2023) used analysis of single nucleotide polymorphisms coupled with phenotypic testing to identify genes associated with acid resistance and acid tolerance in *L. monocytogenes*. A study of 168 *L. monocytogenes* strains identified mutations in the *sigB* operon of several strains that produced acid sensitive phenotypes (Wu et al. 2022). More recently, a similar comparative genomics approach identified a manganese transporter (MntH) as playing a critical role in allowing *L. monocytogenes* to grow at low pH (Wu et al. 2023). In this study, the effect of Mn^2+^ transport was only on growth at low pH but not on survival at low pH, highlighting the fact that the protective mechanisms deployed against acid stress are likely to depend on the precise pH involved, and that acid tolerance and resistance are different phenomena.

Ultimately, it would be ideal if all the responses of individual species to different pH levels could be predicted solely from their genome sequences. While we are still probably a long way from that becoming possible, a machine-learning model has recently been described that allows the prediction of the preferred pH for growth (estimated from relative abundance in environmental samples that cross a known pH gradient) of different bacterial species based on their genome sequence alone (Ramoneda et al. 2023). As more data of this sort becomes available, the ability to predict phenotype from genotype is likely to improve.

#### ‘Omics’ studies: general principles

Beyond the comparative analysis of genome data, the main power of ‘omics-based methods lies in using them to look at the cellular changes at multiple levels (such as RNA, protein, or metabolites) that result from changes in external conditions. Typically in these studies the investigator makes no prior assumptions about what may change, and collects information on everything (to the limit of sensitivity and selectivity of the technique: these limitations are very important and will be discussed in context below) and then uses statistical tools to identify changes by their significance. While this data is of interest in its own right, it is generally used to generate hypotheses about the role and importance of particular genes or pathways, and these hypotheses need to be validated using different methods, such as the construction of individual gene knockouts and analysis of their phenotypes

Omics methods generate very large datasets, but it is important not to mistake quantity or complexity of data for completeness. All methods have limitations in their sensitivity, and to avoid over-enthusiastic use of ‘omics methods where they may not be appropriate, there are additional issues to take into account. First, as careful statistical analysis is at the heart of their study, the use of multiple replicates is essential, although often compromised by the high cost of the methods (triplicates are often used, though there is no sound statistical reason for this particular number). Also because of the very high numbers of data points analysed, corrections to *P*-values are needed to avoid, as much as possible, the danger of false positives. False positives and negatives can never be completely eliminated, however; what is important is to have a meaningful estimate of their level (Fay and Gerow 2013). Second, as interpretation of ‘omics data uses computational tools with complex algorithms at their core, using mathematical methods that are beyond the expertise level of many microbiologists, it is advisable to analyse the same data by more than one tool and compare the outputs, being more suspicious of those that are only given by one of several tools. Third, not all observed changes may be biologically meaningful. There is no *a priori* reason why a change observed under lab conditions necessarily represents something that has been selected for and that has an adaptive function. Care also has to be taken to ensure, as much as possible, that any changes seen are a direct consequence of the test condition. This can be challenging particularly in experiments (the majority) on batch cultures. Large changes occur in the transition between exponential phase and stationary phase, e.g. and if a stress such as low pH imposed on exponential phase cultures causes this transition, many of the changes seen may not be specific to that stress. Fourth, ‘omics data are averages across a population, rather than accurately representing one individual cell, and if the population is heterogeneous, potentially important details about heterogeneity are lost. Fifth, changes at one level are not always reflected at a different level (transcriptional data, e.g. often correlates poorly with proteomics data; Maier et al. 2009, Berghoff et al. 2013, Bathke et al. 2019), so extrapolation of function on the basis of one type of ‘omics data alone is risky. Sixth, most ‘omics data (unless part of a time-course) provide a snapshot; other methods such as the use of promoter probes with reporter proteins whose activity can be followed in real time (discussed above) are much better suited to looking at the dynamics of cellular processes. Finally, it is always important to remember that what is not being measured may nevertheless be key in understanding a particular phenomenon. Studies of mRNA only can miss the role of small RNAs or the impact of differential rates of RNA turnover; proteomics may miss important protein modifications such as phosphorylation or phenomena such as allostery, or may not include some classes of proteins such as membrane proteins; metabolomics levels often do not provide measurements of rates of flux through different pathways that may be more important than levels of individual metabolites.

#### Transcriptomics

Transcriptomics measures the abundance of RNA isolated from cells with a sequence-specific readout, usually giving a figure for the level of expression of all features in the genome [principally open reading frames (ORFs), although estimates of other features such as small RNAs and tRNAs can also be obtained by varying the precise ways in which the RNA is purified]. All methods require initial preparation of total RNA, and because mRNA (particularly in bacteria) is often very rapidly turned over, they usually start with addition to cultures of a reagent that ‘freezes’ the state of the RNA by inactivation of all RNAases. As the majority of RNA in cells is ribosomal RNA, most methods for preparing RNA also include steps for selectively removing it (e.g. by mixing samples with beads coated with sequences complementary to rRNA, which can be subsequently removed), or for enriching the mRNA (e.g. in the case of eukaryotic micro-organisms, by use of the poly-A tails on mRNA for purification on columns or to act as primers in amplification reactions). As the method then produces cDNA from the RNA, either by priming on poly-A tails if present or by using small random hexanucleotides to prime cDNA synthesis, any contaminating DNA has to be removed using Rnase-free Dnases before the cDNA synthesis steps.

Initially, transcriptomic experiments used microarrays, which had a large number (many thousands) of DNA sequences, typically short oligonucleotides or polymerase chain reaction (PCR) fragments corresponding to different positions in the genome, attached at known positions to PVDF membranes or glass slides. In some cases, referred to as tiling arrays, the sequences overlapped each other and covered the entire genome. The choice of sequence of these probes determined what could be detected. Arrays made only of sequences that were complementary to mRNAs made from ORFs predicted from the genome sequences, e.g. would fail to detect antisense transcripts of small RNAs that could hybridize with the transcripts of these ORFs (examples are known where such small RNAs have roles in regulating acid stress responses). Arrays were hybridized with labelled cDNA made from the mRNA that had been prepared from cells grown under specific conditions. Typically labelling was with fluorescent precursors, allowing preparation of two samples from an experimental and a control condition that fluoresced at different wavelengths. These could be hybridized as a mixture to a single array, and high-resolution scanning that allowed detection of each probe hybridizing to each individual spot could be used to give a measure of relative expression levels of each gene under the two conditions.

The first example of the use of this technology to look at a microbial acid response was in *E. coli*, using a commercial array of PCR fragments corresponding to 4290 *E. coli* predicted genes derived from the whole genome sequence (Tucker et al. 2002). Cells were grown to mid-exponential phase in a defined medium at either pH 7.4, 5.5, or 4.5; and RNA was converted to radioactive cDNA and hybridized to the array. The arrays were then imaged, and image processing was used to compare the relative intensities of the spots corresponding to each gene under the different conditions. This process identified the expression of a number of genes as being changed up or down by growth at low pH, including genes that were already known and ones that had not previously been shown to be pH-regulated. To make these designations, the authors had to choose a specific statistical cut-off with regard to both fold-change and *P*-value. Seven genes showing high levels of induction at low pH were chosen for further study, and deletion and overexpression of these showed, perhaps unexpectedly, that only one (designated *yhiE*, later renamed as *gadE* and now known to be a central regulator of the AR2 acid stress response) had a significant effect on the acid resistance profile of the strains at pH 2.75.

This initial study, now over 30 years old, illustrates many of the issues around ‘omics experiments that are still relevant. Its great power was the ability to look in a single experiment at expression levels of the majority of genes, revealing new candidates for study that had not emerged from previous research. It also showed the requirement for statistical analysis (which has become much more sophisticated as the technology has developed), including the need to define a cut-off in order to generate lists of genes whose change in expression is judged to be significant. The conditions of growth had to be specifically defined to obtain reproducible data, with the knowledge that use of different media, different strains, different growth times, and so on, could all produce different gene lists. Strikingly, the magnitude of the change in gene expression was a poor guide as to whether or not loss of the individual genes led to an acid-resistant phenotype, probably at least in part because of functional redundancy within the acid stress response regulon itself, but also possibly related to the poor correlation of transcriptome with proteome.

Array experiments were subsequently done on many microbial species under a range of different pH conditions, including both steady state growth and time-courses of short-term responses to acid shock. From these, many lists of pH-regulated genes were generated, often divided into categories on the basis of similar expression profiles, the subsequent study of which has greatly enriched our understanding of acid stress responses in these organisms. Some studies showed that even different serovars of the same strain could show different responses, highlighting the importance of specifying the experimental conditions precisely (Joerger et al. 2012). Selected examples of these array experiments are given in Table 2, illustrating the range of microbial species studied. But microarray experiments have limitations. The arrays themselves are expensive and specific ones have to be produced for each strain studied. Signal-noise ratios can be high and have to be corrected for, which can reduce the dynamic range of the signal. And arrays are only as good as their design: key features can be missed if complementary sequences to them are not present on the array. For this reason, they have been largely superseded by the more powerful method of RNA-seq.

**Table 2.** Selected examples of transcriptomics studies (microarray and RNAseq) on responses of diverse micro-organisms to low pH, in order of publication.

Organism	Condition(s) (all acidification was with HCl unless otherwise shown)	Method of detection	Citation
*E. coli*	Gene expression analysed following growth at pH 7.4, 5.5, or 4.5 for at least 10 generations	Radioactive cDNA hybridized to PCR-based microarray corresponding to 4290 ORFs	Tucker et al. (2002)
*S. cerevisiae*	Exponential-phase cells stressed with 8 mM sorbate in rich medium	Custom made microarrays containing PCR fragments of 6144 predicted yeast genes	Schüller et al. (2004)
*S. cerevisiae*	20 min growth at pH 4.5 in sorbic acid compared to control at pH 7	Cy3- and Cy5-labelled cDNA hybridized to PCR-based microarray corresponding to 6144 ORFs	Schüller et al. (2004)
*S. aureus*	Time course of response to change in pH from 7.3 to 4.5 (2, 5, 10, and 20 min postacidification)	Cy3- and Cy5-labelled cDNA hybridized to PCR-based microarray corresponding to 2693 ORFs	Bore et al. (2007)
*S. cerevisiae*	Transcriptional responses to four weak organic acids (benzoate, sorbate, acetate, and propionate) were investigated in anaerobic, glucose-limited chemostat cultures.	*S. cerevisiae* affymetrix DNA chips	Abbott et al. (2007)
*E. coli*	Time course of response to change in pH from 7.6 to 5.5 (0, 1, 5, and 10 min postacidification)	Biotin-labelled cDNA hybridized to customized affymetrix tiling array	Kannan et al. (2008)
*A. niger*	Growth in bioreactors at three different pH setpoints pH 2.5, 4.5, and 6.0	Customized affymetrix microarray(3AspergDTU)	Andersen et al. (2009)
*S. mutans*	Comparison of expression following 2 h growth at pH 7.5 and 5.5	AlexaFlour 555- and 647-labelled cDNA hybridized to array of 70-mers corresponding to 1948 ORFs	Gong et al. (2009)
*S. cerevisiae*	Exponential-phase cells exposed to acetic acid (at pH 4); propionic acid (at pH 4)	*S. cerevisiae* affymetrix DNA chips	Mira et al. (2009, 2010a)
*B. subtilis*	Comparison of expression following growth from 1:500 dilution to OD_600_ 0.2 at pH 6, 7, or 9	Biotin-labelled cDNA hybridized to custom affymetrix tiling array	Wilks et al. (2009)
*Lactobacillus casei*	10 and 20 min incubation in media at pH 6.0, 5.0, 4.8, 4.5, 4.3, 4.0, 3.8, 3.5, or 3.0	Customized affymetrix microarray covering 2691 ORFS	Broadbent et al. (2010)
*Salmonella enterica*	Two different serovars incubated for 10 min at pH 6.8, 5.5, 5.5 + acetic acid, and 4.5	Cy3- and Cy5-labelled cDNA hybridized to PCR-based microarray corresponding to ORFs from multiple *Salmonella* species	Joerger et al. (2012)
*L. monocytogenes*	Growth from OD_600_ 0.4 to 1.2 in 10 mM acetic acid, 20 mM lactic acid, or hydrochloric acid, all at pH 5, with pH 7 control	Fluorescent cDNA hybridized to PCR-based microarray	Tessema et al. (2012)
*C. glabrata*	Exponential-phase cells cultivated in the presence of 1 mM sorbate	Custom made *C. glabrata* agilent microarray	Jandric et al. (2013)
*Ustilago maydis*	Cultures grown in liquid minimal medium at pH 7 or pH 3 to obtain the yeast or mycelium forms, respectively.	High density (one color) arrays from NimbleGen Microarray covering 6883 genes	Martínez-Soto and Ruiz-Herrera (2013)
*C. albicans*	Overnight-cultivated cell exposed to HCl, lactic, acetic, propionic, or butyric acids	RNA-seq on cDNA prepared libraries	Cottier et al. (2015)
*C. glabrata*	Overnight cultures grown at pH 4 that were suspended for 1 h at either pH 4 or 8	RNA-seq of total RNA	Linde et al. (2015)
*S. cerevisiae*	Exponential-phase cells exposed to HCl, acetic acid or lactic acid	Custom made DNA microarrays	Kawahata et al. (2015)
*E. coli*	Growth of cultures to OD0.3 at pH7 or pH 5.5	RNA-seq of total RNA following removal of 16S and 23S rRNA	Seo et al. (2015)
*Penicillium expansum*	Fungal cultures grown under pH 4 or 7	RNA-seq of total RNA	Barad et al. (2016)
*C. glabrata*	Exponential-phase cells cultivated in the presence of 30 mM acetic acid	Custom made *C. glabrata* Agilent microarrays	Bernardo et al. (2017)
*Bacillus megaterium*	Comparison of expression following 4 h growth at pH 7 or 4.5	RNA-seq of total RNA following removal of 16S and 23S rRNA	Goswami et al. (2018)
*Rhizobium tropici*	45 min incubation of mid-log phase cells at pH 6.8 or pH 4.5	RNA-seq of total RNA	Guerrero-Castro et al. (2018)
*E. coli*	Comparison of expression under balanced growth conditions at pH 7 or pH 5.5, aerobic or anaerobic, with addition of acetic, propionic, or butyric acid	RNA-seq of total RNA following removal of 16S and 23S rRNA	Bushell et al. (2020)

RNA-seq at its simplest involves sequencing all the RNA molecules present in a sample (although mRNA or small RNAs are often enriched first, as described above), by converting the RNA to cDNA and using ‘next generation’ sequencing methods, on one of the many different sequencing platforms now available. The sequence data generated usually consists of millions of short ‘reads’ (typically between 50 and 200 bases). Output files containing this raw sequence data are then processed through a series of bioinformatic steps, referred to as a ‘pipeline’, to remove low quality reads and to align the remaining reads against the genome, the ultimate aim being a read-out of levels of expression of all transcribed features in the genome. Usually, this is a number that represents the frequency of reads corresponding to the transcript of a given gene relative to the total number of transcripts, corrected for gene length, and is often expressed as RPKM (reads per kilobase per million reads that have been mapped to the genome).

This simple description hides a mountain of detail about the care with which RNA-seq experiments need to be designed, executed, and interpreted if they are to yield high quality information (Creecy and Conway 2015). In particular, as the tools to analyse high throughput data have grown ever-more sophisticated, the input of expert bioinformaticians should be sought before any experiments are undertaken, and their assistance in the interpretation of the data output may be invaluable. The development of databases of gene ontologies (where genes are classified according to their molecular function, the cellular location of their products, and the biological processes in which they are involved) means that all transcriptomics data can now give information not only about expression of individual genes but also about the pathways and global processes in which they are implicated, thus giving us a high-level view of events in the cell. This is particularly the case if RNA-seq results are coupled with the outputs of other high throughput methods, such as global detection of binding sites of regulator proteins involved in the acid shock response: in such cases, transcriptome data is usually gathered under acid stress conditions in the wild-type strain but also in selected strains where different regulator proteins have been deleted (Aquino et al. 2017). RNA-seq has a higher dynamic range than the microarrays that it has largely replaced, and it does not require customized arrays for each strain studied. However, the hypotheses generated from the large amounts of data that RNA-seq produces still need to be validated, often by the time-honoured method of constructing individual mutant strains and analysing their acid resistance profiles. It also needs to be borne in mind that RNA-seq has to date largely been used as a population level technique, where individual cell-to-cell variation (increasingly being regarded as biologically important) is not captured. Methods for RNA-seq on single cells are however becoming available (Homberger et al. 2022). A recent study on *E. coli* cells in early stationary phase using single-cell RNA-seq was able to demonstrate the existence of a small subpopulation of acid-resistant cells, initially identified because of the high level expression of some of the genes associated with the acid stress response (Wang et al. 2023). Examples of some RNA-seq experiments that have been done looking at different aspects of acid stress are listed in Table 2.

#### Proteomics

Another commonly used ‘omics method, proteomics, is described next. Knowing the nature and levels of proteins in the cell may give more accurate insights into cellular processes than transcriptomics does, particularly given the generally low correlation between mRNA and protein levels. However, proteomics data are less comprehensive, with lower limits of detection, than transcriptomics, and can be technically more challenging to generate, particularly if post-translational modifications (which can significantly affect activity) are to be determined.

Proteomics methods aim to detect and quantify all proteins synthesized under control and test conditions, and to identify those that show a significant change in level between the two conditions. A knowledge of the complete genome sequence of the organism being studied is generally required, as identification is mostly done by matching peptides produced by digesting the proteins with a theoretical database of all possible peptides that would be produced if the entire proteome were to be digested with the same protease,. Because the number of possible peptides produced will be very high, these methods rely on measuring, with the highest possible precision, the molecular masses (strictly, the mass/charge ratio) of the peptides detected, a process that requires mass spectrometry. As with other ‘omics tools, biological replication is needed to generate statistical estimates of the significance of the change in protein level, which is generally used to generate lists of proteins that are then studied more closely.

Many variants of proteomics begin by separating the proteins in the sample. Classically this was done using 2-dimensional gel electrophoresis (2-DGE), separating proteins by charge in the first dimension and by mass in the second. Proteins can be labelled and visualized by autoradiography, or directly stained for visualization; in all cases, a range of scanning methods are used for quantification. Early methods used N-terminal analysis of spots eluted from such gels to identify the proteins concerned (e.g. Blankenhorn et al. 1999), but various different MS methods are now used more or less exclusively, usually on peptides after digestion of the protein spots, often while still in the gel. Separation of proteins is now more often done in a preliminary liquid chromatography step, followed by MALDI-TOF (matrix-assisted laser desorption/ionization time-of-flight) or MS/MS (an example of tandem mass spectroscopy, where two separation steps coupled with fragmentation of ionized peptides after the first separation increase the accuracy of measurement). The details of these methods and their many variants have been reviewed extensively elsewhere (e.g. Intelicato-Young and Fox 2013). Examples of proteomics studies on aspects of acid stress are shown in Table 3; the conditions chosen for the application of the acid stress in these studies were sometimes based on those expected to be encountered in food processing. Another important development in proteomics is the development of tags (stable isotopes or specific chemicals, in methods known as SILAC and ITRAQ, respectively) that are used to label samples in advance, after which they can be mixed with separation of signals being done in the data processing step. These tags enable quantitative analysis of mixed protein samples (Xia et al. 2016, Zhang and Lyu 2023).

**Table 3.** Selected examples of proteomics studies on responses of diverse micro-organisms to low pH, in order of publication.

Organism	Condition(s) (all acidification was with HCl unless otherwise shown)	Method of detection	Citation
*E. coli*	1:100 dilution of overnight culture, then growth in neutral, acidic (pH 4.5), neutral or basic media to OD600 0.35	2D-GE gels scanned and quantified; spot densities compared. Selected spots eluted and N-terminally sequenced to identify protein.	Blankenhorn et al. (1999)
*L. monocytogenes*	2 h growth of mid-log phase bacteria at pH 7.2 or 5.5, or treatment at lethal pH (3–3.5)	^35^S-met-labelled samples separated by 2D-GE and visualized. Selected spots cut out, trypsinized *in situ*, peptides identified by MALDI-TOF	Phan and Mahouin (1999)
*Acidithiobacillus caldus*	Cells grown in continuous culture at pH 2.5 and acidified with H_2_SO_4_ to pH 1.1; harvested at steady state.	Samples separated by 2D-GE and visualized. Selected spots cut out, trypsinized *in situ*, peptides identified by MALDI-TOF	Mangold et al. (2013)
*Lactococcus lactis*	Exponential phase cells resuspended in media at pH 7, 5, or 5.5 for 2 h	35S-met-labelled samples separated by 2-DGE and visualized. Selected spots cut out, trypsinized *in situ*, peptides identified by MALDI-TOF	Budin-Verneuil et al. (2005)
*L. monocytogenes*	Cells grown to OD 0.4 incubated for 1 h at pH 4.5.	Comparison of quantification of gels between wild-type and *sigB* mutant, excision and digestion/MALDITOF	Abram et al. (2008)
*Trichoderma reesei*	Cultivation of different strains secreting hydrolytic enzymes were cultivated at pH 3.0, 4.0, 5.0, 6.0, 7.0, 8.0, and 9.0	Analysis of the secretome by SDS-PAGE followed by tryptic digest analysis by LC–MS/MS	Adav et al. (2011)
*L. monocytogenes*	Progressive addition of HCl and lactic acid to growing culture, samples taken at 5, 7, 8, and 15 h	Protein extracts digested with trypsin, peptides identified using 2D-LC-MS/MS	Bowman et al. (2012)
*Shigella flexneri*	1:20 dilution from overnight culture; then 5 h growth at pH 7 or 5	SDS-PAGE, cut into 1 mm^3^ cubes, digest in gel with trypsin, nanoflow LC-MS/MS	Yu et al. (2017)
*Oenococcus oeni*	Cells incubated in growth media at different pH values (4.8, 4, 3.5, and 3.2) for 2 h	Proteins separated by 2D-GE, selected spots identified by in gel digestion and MALDI-TOF/TOF	Yang et al. (2021)
*Campylobacter jejuni*	Stationary phase cells incubated for 2 h at pH 4, or grown for ca. 20 h at pH 5.8, 7, or 8	Protein extracts digested with trypsin, peptides identified using LC-MS/MS	Ramires et al. (2023)

As an illustration of the effectiveness of these methods and the ongoing development of the technology, while also emphasizing the care with which they need to be interpreted, it is interesting to look at proteomic experiments on one particular species, *L. monocytogenes*. This major food-borne pathogen has an inducible acid stress response that helps it to survive at very low pH, and there are concerns that the relatively mild acid conditions that it can be exposed to in some foods might induce high levels of resistance that would enable it to survive gastric acid. Thus, there have been several proteomic studies of this strain at low pH, with and without other stresses (Phan and Mahouin 1999, Abram et al. 2008, Bowman et al. 2012, Melo et al. 2013a,b, He et al. 2015). These have been done on a range of laboratory strains and different food and clinical isolates. The conditions used have varied from spinning and resuspension of actively growing cultures in media of different pH values for different lengths of time, to stepwise acidification with both inorganic and organic acids. Other than acid stress being a common factor, there is little overlap in the experimental parameters used in these different studies, and it is perhaps therefore unsurprising that there are large differences in the proteins identified in the different studies, as well as some overlaps. Indeed, even in those studies where different strains of *L. monocytogenes* were compared under identical conditions, large strain-specific differences were seen in their response to low pH (Melo et al. 2013a,b). This again emphasizes the need to be very precise in the description of all conditions used in acid stress experiments, and to be cautious in extrapolating from the data obtained, particularly in the absence of any further validation of the role of individual genes.

#### Metabolomics

The metabolome is defined as all the small molecules that together are components of the cell’s metabolic pathways. Determination of the metabolome can give insights into how cells respond to stresses or different growth conditions by favouring some metabolic pathways over others. A knowledge of the genome sequence of the organism is not required for the metabolome to be determined, although in practice metabolomes are often analysed alongside proteome and transcriptome data, which do require such knowledge. The metabolome is very dynamic, with many small molecules having half-lives of seconds or less, so capturing an accurate snap-shot of the state of the metabolome (particularly following a stress-induced change) requires very rapid sampling combined with treatment to quench all activity in the sample. Metabolomics may be targeted, where one specific set of metabolites is examined (e.g. lipids), or untargeted, where the survey of metabolites is as comprehensive as possible. The majority of studies use a combination of liquid or gas chromatography of samples to achieve a degree of separation of the components followed by mass spectroscopy (Kuehnbaum and Britz-McKibbin 2013). NMR can also be used, which does not require a prior separation step, but it is less sensitive. For example, studies have demonstrated the application of metabolomic analysis in revealing time-dependent responses of microbial communities to dynamic environments and their responses to various stress conditions (Nugroho et al. 2015). The application of metabolomics for investigation of acid stress response of microorganisms involves culturing microorganisms under specified conditions (e.g. acidic conditions), extraction of intracellular and/or extracellular metabolites depending on the particular aim of the study, separation and identification of metabolites by using analytical techniques including mass spectrometry, gas chromatography–mass spectrometry, or nuclear magnetic resonance spectroscopy, and computational analyses of the data for identification of specific metabolites that are upregulated or downregulated in response to acid stress (Aguiar-Pulido et al. 2016, Balluff and McDonnell 2018, Lamichhane et al. 2018). In this way, the metabolic pathways that are involved in or affected by growth at low pH can be identified.

Metabolomics studies are in general less widely used in research on acid responses in micro-organisms than the other ‘omics methods described above, but there are examples in the literature. A particularly comprehensive study integrated transcriptomics, proteomics, and metabolomics data on *Sinorhizobium meliloti* cells grown in chemostats at either pH 7 or 6.1 (Draghi et al. 2016). In this study, GC–MS was used to separate metabolites, with the output peaks being assigned to specific metabolites by reference to standards and to online databases of known metabolites. A tpotal of 19 metabolites showed significant increases or decreases between the two conditions, and this information was used, together with other ‘omics datasets, to infer changes in the extent to which different metabolic pathways are active under the different growth conditions. A limitation of the study, acknowledged by the authors, was that it only looked at the cytoplasmic metabolome; a fully comprehensive study would include compounds that are secreted by the cell, as is often a consequence of overflow metabolism.

Such a study has been taken looking at the metabolic consequences of acid stress on *E. coli* under different growth conditions, with cultures grown in minimal media at pH 7 or 4.5 with glucose as a carbon source plus a range of different added amino-acids, in an attempt to activate the different known acid resistance mechanisms of *E. coli* (Gold et al. 2022). This study looked specifically at both polar metabolites and at lipids, which require different methods of extraction, using LC-MS to analyse both, and concluded that the majority of significant changes in polar metabolites that correlated with different supplements were in the external metabolome, rather than the cytoplasmic metabolome. The study also showed the relative distribution of different lipids was dependent on the different growth conditions. In another recent study, Liu et al. (2021) integrated metabolomics with transciptomics to investigate the impact of environmental stresses, including low pH, oxidative stress, and bile stress on the mechanism of *Bifidobacterial* biofilm formation. Their findings showed the importance of quorum sensing, two component systems, and amino-acid metabolism during biofilm growth.

Other studies reporting changes in metabolites associated with acid stress have appeared in the literature (e.g. Shayanfar et al. 2018, Wang et al. 2022) including a study using NMR (Chen et al. 2020a) but the technical complexities of metabolomics and the challenges in linking the complex datasets to a better understanding of the underlying biology means that this is a field that is still relatively undeveloped.

#### Other postgenomic methods for identifying genes with a significant role in acid resistance

The poor correlation that is often found between different ‘omics methods raises the question of whether the more classical genetic approaches had it right all along: is the isolation of mutants with acid sensitive or acid inducible phenotypes the best way to quickly identify key players in the acid stress responses of different organisms? One problem is that such isolation can be laborious. As we saw earlier, random mutagenesis requires lengthy screening to identify mutants of interest. An alternative reverse-genetics approach, made possible by the availability of whole genome sequences, is to begin with large numbers of known mutants and test them systematically for their phenotype at low pH. For example, arrayed libraries of all nonessential gene deletions are now available for many model organisms, and these have been profiled for growth by spotting onto media containing different organic acids or at low pH. Such large-scale studies have been used to identify the *S. cerevisiae* genes and pathways mediating tolerance to low pH, including in the presence of important organic acids (Mollapour et al. 2004, Kawahata et al. 2006, Mira et al. 2009, 2010). Alternatively, transposons can be used to generate large numbers of random mutants for screening, a method that continues to be valuable now that the availability of whole genome sequences makes the identification of the tagged genes quite straightforward (e.g. by using inverse PCR with transposon-specific primers to identify the sequence into which the transposon has become inserted; Riccillo et al. 2000, Madeo et al. 2012, Vivjs et al. 2016, Palud et al. 2018). A refinement of this approach is to grow very dense transposon libraries (sometimes with over a million different insertions sites in the genome, created by pooling large numbers of unique independent colonies) under different conditions, and to use high throughput sequencing of PCR-generated fragments that contain the transposon insertion site to estimate changes in the frequencies of strains carrying mutations in different genes after such growth. If these frequencies decline under a stress condition, it suggests the gene concerned contributes to the fitness of the strain under that condition, a hypothesis that can be tested by constructing strains with deletions of the genes concerned and competing them against the wild type parent under the stress condition. This method (usually referred to as Tn-seq or TraDIS) has become widely used in bacterial genetics in the last decade (Cain et al. 2020), although studies where it has been used to look at the effects of low pH stress are still relatively rare (Rau et al. 2016, Bushell et al. 2020, Gu et al. 2021, Mandall et al. 2021, Liu et al. 2023). A drawback of Tn-seq/TraDIS is that it cannot provide any readout for essential genes, as by definition mutants in these cannot be made, whereas changes in level of expression of such genes can be seen via transcriptomic or proteomic methods. An alternative approach is to use CRISPRi libraries, which express guide RNAs targeting the promoters of genes selected by the investigator in the organism under study, together with a modified Cas9 protein that binds to, but does not cleave, the DNA defined by these guide RNAs. This has the effect of inhibiting gene expression, and as expression of the guide RNAs can themselves be turned on or off, and the level of inhibition can be fine-tuned by appropriate choice of guide RNA, essential genes can also be studied using this method. This approach has been used to define genes important in tolerance to different organic acids in *S. cerevisiae* (Mukherjee et al. 2021, 2023).

A different approach to identify genes of importance is to use laboratory-based evolution. As a general approach, laboratory-based evolution has been around for many years, but has become particularly popular now that the speed and low cost of whole genome sequencing has made it easy to very rapidly identify mutations in evolving populations (Conrad et al. 2011, Dragosits and Mattanovich 2013, van den Bergh et al. 2018). The basic idea is simple: several replicate populations of micro-organisms are exposed to a specific stress over a long period of time, typically weeks to months, either by long-term growth in bioreactors or chemostats, or more commonly by repeated growth and dilution. Over time, mutations that enhance the fitness of the organism under these conditions are expected to arise by random chance and these will become more predominant in the population due to natural selection, as long as they are not lost when the population passes through bottlenecks at the dilution step. The consequences may be seen by an improving growth rate over time of the evolving population under the stress condition. Whole genome sequencing can then identify the mutations that have arisen in the populations under question. It is often the case that mutations affecting the same genes are repeatedly found in independent experiments, although the specific mutations differ. Samples of the population can be taken and frozen at any point, thus creating a ‘fossil record’ that enables the order of appearance of mutants and hence the specific evolutionary pathway to be determined. Mutations that are candidates for leading to improvements in fitness can also be found by comparison with the genome sequences of strains evolved in parallel under nonstress conditions, and these candidates can be introduced into the original wild-type background to test their phenotypic effects. This approach has been used as a tool to study the process of evolution itself (Blount et al. 2018), but also to gain insights into the genes and pathways that can contribute to improved fitness under different acid stress conditions, as well as to engineer industrial strains where improved survival under low pH is needed. Some examples of these studies are given in Table 4.

**Table 4.** Examples of studies using laboratory-based evolution to explore responses to acid, in order of publication.

Organism	Selective condition	Citation
*E. coli*	Repeated exposure to short lethal pH	Johnson et al. (2014)
*E. coli*	730 generations of growth at pH 4.8, followed by 1270 generations at pH 4.6	Harden et al. (2015)
*Leuconostoc mesenteroides*	Long-term growth (1 year) in media with stepwise increases in lactic acid concentration	Ju et al. (2016)
*E. coli*	800 generations of growth at pH 5.5	Du et al. (2020)
Haloarchaeon *Halobacterium* sp. NRC-1	500 generations at pH 6.3 (other stresses also tested)	Kunka et al. (2020)
*S. cerevisiae*	Testing tolerance to coumaric acid and ferulic acid at a pH of 3.5	Pereira et al. (2020)
*S. cerevisiae*	pH was decreased from 3.0 to 2.5, in 70 generations	Tian et al. (2020)
*Ralstonia solanacearum*	1500 generations of growth at pH 4.9	Liu et al. (2022)
*Oenococcus oeni*	560 generations with step-wise reduction from pH 5.3 to 2.9	Julliat et al. (2023)

## Acid resistance, acid tolerance, and the acid stress responses in the natural environment

### Specific issues around isolation and study of extreme acidophiles

Most of what has been discussed above refers to studies on neutralophiles: organisms that grow optimally at around pH 7. However, for researchers interested in acid stress responses, there are excellent reasons for also studying acidophiles. First, their adaptations for growth at low pH can inform us about the strategies used to combat low pH and the ways in which niche-specific resistance can evolve (and can potentially be engineered *de novo* in less acid-tolerant organisms). Second, they have applications in areas such as bioremediation and bioleaching in very acidic environments, and further potential will only be realized if their underlying biology is understood. For this reason, we will now review some of the methods that can be used for isolation, cultivation, and study of acidophilic micro-organisms.

Micro-organisms living under acidic conditions are usually divided into two main groups—moderate acidophiles with optimal growth at pH around 5 and extreme acidophiles that grow optimally at pH 3 or lower. Both extremely acidic natural and anthropogenically made environments contain relatively low amount of organic carbon, which means that organisms living in these environments face not just low pH but also low carbon content. Other stresses in these environments can include high concentrations of dissolved metal ions and varying temperature. As such, the acidophiles are a very heterogeneous group (Tran et al. 2017, Quatrini and Johnson 2018b, Saralov 2019). The strategies they use to maintain near-neutral pH, despite the high proton gradient across their cytoplasmatic membrane (Hu et al. 2020, González-Rosáles et al. 2021), include mechanical defence, proton efflux, intracellular buffering, and repair or protection of biomacromolecules (Liu et al. 2015, Bellenberg et al. 2019). In particular, unlike neutrophiles, acidophiles have an inside-positive membrane potential that creates an electrochemical barrier to proton influx (Slonczewski et al. 2009, Buetti-Dinh et al. 2016, Rivera-Araya et al. 2019). Mechanisms necessary to maintain pH homeostasis have been found together with mechanisms that give high tolerance to cations (Massello and Donati 2021, Sedláková-Kaduková 2022). However, high concentrations of anions (except sulphate) in their environment are toxic to acidophiles (Rivera-Araya et al. 2019). All these poly-resistance mechanisms make them very versatile living in extreme environments (Massello and Donati 2021). On the other hand, the extent of how these strategies contribute to acid tolerance at the population level is still not fully understood (Quatrini and Johnson 2018b).

**Figure 4.**
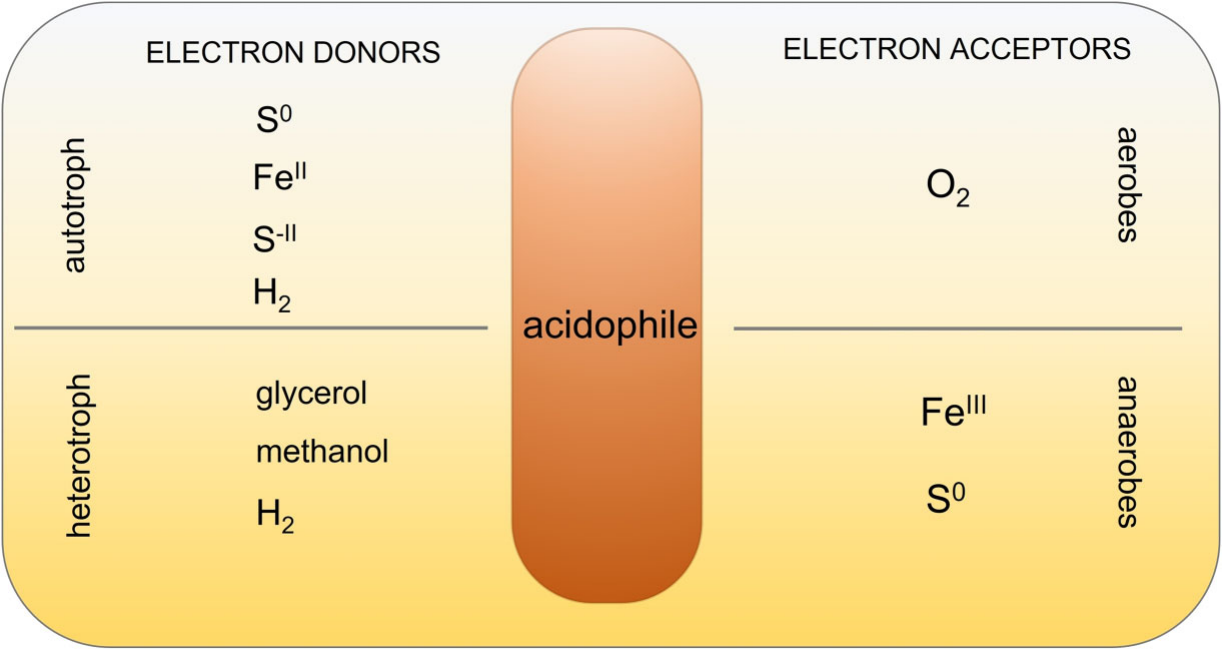
Illustration of the different types of acidophiles and the electron donors and acceptors that they use

Acidophiles can utilize different sources of energy (solar, organic, and inorganic compounds) and different compounds as electron acceptors (oxygen, ferric iron, and sulphur; see Fig. 4). Autotrophy, heterotrophy, and mixotrophy have all been documented among acidophiles. One very typical feature of micro-organisms living in these environments is an ability to grow using inorganic electron donors (chemolithotrophy), e.g. Fe and S (Zhang et al. 2013) or As (Lebrun et al. 2003). The most studied extremely acidophilic bacteria are members of the genus *Acidithiobacillus*, Gram-negative chemolitotrophic bacteria able to grow between pH 0.5 and 5 with an optimum of 3 (Johnson 2018). All of them can utilize reduced sulphur compounds as electron donors, and some but not all can use ferrous ion (Falagán et al. 2019). Utilization of hydrogen as a sole electron donor is a common trait for some species but not others (Norris et al. 2020).

Based on these points, it is obvious that one of the most important factors affecting the isolation and cultivation of extreme acidophiles is the composition of growth media. The choice of compound to act as the electron donor (energy source) is particularly important. Massello and Donati (2021) found that the culturing process shaped the phylogenetic structure and functional potential of acidophilic consortia more strongly than presence of elevated concentrations of various metal ions.

Correct media are also important in the efficiency of applications using acidophiles. For example, utilization of minimal media with low sulphate concentration and absence of some nutrients leads to higher final removal of inorganic sulphur during a coal desulphurization process assisted by *A. ferrooxidans* alone or by or a consortium of *A. ferrooxidans* and *A. thiooxidans* (Duarte Briceno et al. 2020). The iron oxidation state in media affects bioleaching as it can act as an electron acceptor or electron donor. As an electron donor it will support bioleaching of metals that need to be oxidized (e.g. Ni, Cd from Ni–Cd batteries, or metals from printed circuit boards) and contrariwise as an electron acceptor it will support bioleaching of metals that need to be reduced (e.g. Mn from alkaline batteries or Co from Li accumulators) (Sedláková-Kaduková et al. 2017).

Cultivation with particular energy sources can also change the response to particular stresses. For example, cultivation of *A. ferrooxidans* in iron(II)- or sulphur-containing media resulted in higher sensitivity of cells towards oxidative stress in comparison with cell cultivated on pyrite (Bellenberg et al. 2019). Cultivation of acidophiles in specific media (e.g. missing some important mineral nutrient) also can help to understand their physiology. For example, sodium and/or potassium ions in media are important for maintaining pH homeostasis, as cultivation of extreme acidophiles in media lacking these resulted in a reduced growth rate and increased sensitivity to pH changes (Buetti-Dinh et al. 2016).

The most commonly used medium for chemoautotrophic acidophiles is a 9-K medium first described by Waksman and Joffe (1922) with iron sulphate as the energy source. The composition of 9 K medium can be altered according to the specific needs of specific organisms. The most common additional compounds are yeast extract (Johnson et al. 1987), sulphur with a purity higher than 99.5% (Liu et al. 2020), and pyrrhotite (Chen et al. 2008). The medium can be modified so that the only source of energy is another metal, e.g. As(III) (Sehlin and Lindström 1992, Goltsman et al. 2009). Further details of how to prepare these media can be found elsewhere (Pogliani et al. 1999, Goltsman et al. 2009, Chen et al. 2009, Gan et al. 2015, Valdebenito-Rolack et al. 2017). In addition to 9 K medium, sterile autotrophic basal salts medium can also be used for the cultivation of some chemoacidophilic bacteria (*A. ferrooxidans*). Due to the aerobic metabolism of chemoacidophilic bacteria, it is important to maintain a good oxygen supply during isolation and cultivation (Lavalle et al. 2005, Arshadi et al. 2021). Similarly, cultivation of heterotrophs from extremely acidic environments depends on the correct selection of the main carbon/energy sources. Using organic acids may lead to unsuccessful isolation as at low pH value these acids shift towards their lipophilic nondissociated forms, which can diffuse into the cells and then dissociate, lowering the intracellular pH. According to Holanda and Johnson (2020) nonionic substrates such as glycerol, hydrogen, and methanol are more suitable electron donors for heterotrophs at very acidic pH. More information on suitable media for acidophilic and acid-tolerant sulfate reducing bacteria are summarized in Nancucheo et al. (2016).

The most common isolation techniques for acidophiles use liquid media. Agar plate cultivation cannot be applied easily and for many years was considered not possible, partly because agar in acidic medium is easily hydrolysed and fails to form a solid surface. Application of gerlite (polysaccharide derived from bacteria) as a gelling agent improved thermostability of solid media under higher temperatures (over 50°C) and has enabled plate cultivation of some thermophilic acidophiles (Romano et al. 1991). There are also possibilities to prepare plates at low pH (at least down to pH 2.5) using short heating times by microwaves, or by preparing agar stock independent of a buffered salt solution and mixing after autoclaving (Kanazawa and Kunito 1996, Lucena et al. 2020). Besides various media modifications, other techniques such as overlay technique have been applied using cocultivation of autotrophic and heterotrophic acidophiles. Toxic compounds produced in the plate by hydrolysis were removed by heterotrophic acidophilic species facilitating the growth of autotrophic species (Johnson 1995, Nancucheo and Johnson 2020). The overlay technique uses a double-layered gel in a standard Petri dish. The lower layer is inoculated with a heterotrophic acidophilic culture, e.g. *Acidiphilium* sp., and the upper layer is used for cultivation of autotrophic acidophiles. Heterotrophic acidophile degrade small organic molecules produced during acid hydrolysis of agar and allow the growth of chemolitotrophic bacteria on solid media (Johnson 1995). Another way to overcome the challenges related to agar plating is by cultivation on membrane filters. Here, membrane filters normally used for filtering liquid media are directly placed on agar surfaces avoiding the contact of bacteria with agar. Floating polycarbonate membranes have also been used for successful acidophile cultivation, eliminating the need of solid medium (Johnson and Hallenberg 2007).

Bacteria that are able to survive and grow under a large pH range have a high potential for various biotechnological applications. However, their isolation is specific as these bacteria can be isolated with acidophiles as well as neutrophiles. To distinguish them, significant effort is necessary using sequential cultivations starting with media for neutrophiles following with acid media. To obtain a pure culture requires a considerable amount of time and effort. Takano and Aoyagi (2022) recently developed a specialized cellulose film cultivation method to isolate the acid-tolerant bacteria in a single step. In this method, a cellulose film covers a nonwoven fabric soaked in liquid medium. Colonies form on the cellulose film surface and can be easily transferred from one nonwoven fabric to another, both soaked with different media. The advantage is that total number of isolates is reduced as nonacid-tolerant bacteria are excluded immediately during the cultivation.

A step following isolation where cultivation conditions are very important is a period of adaptation. Although natural consortia isolated from ores serve as a basis for biomining studies, they are not as efficient as adapted ones. Adaptation takes between days to several weeks. During this period, consortia become adapted to target materials (ores, concentrates, or waste) resulting in significant increases of efficiency and reduction in retention times (Rawlings 2007, Mražíková et al. 2013). This process is sometimes called ‘domestication’ as it describes the artificial selection of native species to obtain cultivated variants with desirable characteristics (Gallone et al. 2018). Little is known about the genetic basis of this process for extreme acidophiles, but one metagenomics study showed it was linked to a reduction in the diversity of the consortium, with a few species becoming dominant (Ulloa et al. 2019).

### Population-level studies: heterogeneous environments and populations, and the complexities of dealing with multiple variables

Up to this point, we have largely been considering experimental set-ups that involve pure cultures of single species. However, there are strong reasons for also wanting to study mixed communities, which are more representative of the ways in which micro-organisms grow in natural environments, and that also may be specifically engineered for biotechnological applications. Mixed microbial communities, also known as heterogeneous/multispecies groups, are made up of multiple species coexisting and interacting with each other in natural or designed environments (Gorter et al. 2020), and they show different interactions ranging from mutualism to competition. Such communities play a crucial role in biogeochemistry in nature, as well as bioremediation and treatment of contaminated water, soil, and air. However, due to the limitations of cultivation techniques, many metabolic processes in mixed microbial communities remain unknown. To understand them better, experiments may be done by sampling and analysing directly from natural environments, or by mimicking nature in microcosm/mesocosm experiments. Engineered mixed populations deliberately engineered for purposes such as using bioreactors for treating wastewater or for biobased production, have also been studied. Above, ‘microcosm’ refers to small, artificial open or closed ecosystems that simulate natural conditions, while ‘mesocosm’ refers to larger, artificial, and mostly outdoor systems. They are distinct from bioreactors, which are designed containers where biochemical reactions occur, and operational and environmental parameter are fully controlled.

A sampling strategy to analyse mixed microbial communities in ecosystems (whether as microcosms, mesocosms, natural, or engineered) is necessary to reveal population dynamics, and to deal with the complexity of the mixed microbial community and its response to changing conditions.

### Environmental and operational conditions

Various studies have examined microorganisms' acid stress responses at different population levels in different ecosystems (Zanotti and Cendron 2010, Guan et al. 2013, Jain et al. 2013). Many variables can influence such studies, particularly in heterogeneous environments and populations, where multiple factors are at play. Specifically, pH level, temperature, genetic variability, nutrient availability, and microbial interactions can all impact population–level investigations of acid stress response in heterogeneous environments (Guan and Liu 2020, Lund et al. 2020). Below, we consider some of these in turn.

#### pH

In controlled environments such as bioreactors, the acid stress response of microorganisms can be readily monitored and the pH tightly controlled. Microorganisms employ different strategies to cope with acid stress at different pH values, and understanding these mechanisms is critical for optimizing the production capabilities of industrial strains in bioreactors. For example, acidic pH (pH 5) has been shown to improve volatile fatty acids (VFAs) production in long-term reactor operation: the VFA production yield (gCOD/gVS) was 0.92 under pH 5 but only 0.42 under pH 10 and 0.21 under neutral pH (pH 7) (Atasoy and Cetecioglu 2022). Thus, controlling the pH of the bioreactor is essential for maintaining microbial viability and productivity and achieving the desired outcome of the bioprocess.

The pH levels in natural environments, however, cannot be experimentally adjusted, and vary greatly. The pH of aquatic ecosystems, such as oceans, lakes, and rivers, can be influenced by many natural factors, such as weathering of rocks and minerals, and human activities, such as industrial pollution (Bashir et al. 2020). Ocean acidification consequent to raised CO_2_ levels is a global issue that significantly affects marine environments and microbial diversity. As a result of ocean acidification, microbial diversity in marine environments and the activities of microbial communities are significantly affected (Das and Mangwani 2015). Microcosms can be used to some extent to model this; e.g. a study mimicking a marine bacterial community showed that changes in pH shifted the bacterial community profile in terms of its members and their relative abundances (Krause et al. 2012).

Monitoring of acid stress responses in ecosystems can involve the assessment of multiple indicators and parameters to discern the consequences of acidification on organisms and ecosystem functioning. This includes (a) monitoring changes in environmental pH; (b) utilizing acid-sensitive organisms as bioindicators; (c) monitoring chemical parameters such as alkalinity, calcium concentration, sulphate levels, or total dissolved inorganic carbon to evaluate water’s buffering capacity and identify sources of acidity; (c) employing biomarkers, including enzyme assays, protein analysis, or gene expression profiling, to investigate molecular or cellular indicators of acid stress; (d) assessing overall ecosystem functioning by monitoring rates of nutrient cycling, decomposition, and other ecosystem processes to reveal acidification-induced alterations, and (e) conducting controlled experiments, such as mesocosm studies, to observe the responses of specific organisms or communities under controlled conditions. By employing this multifaceted approach, a comprehensive understanding of the impact of acidification on ecosystems can be attained.

Terrestrial ecosystems, such as forests, wetlands, and deserts, also have varying pH, influenced by factors such as soil type, precipitation, and vegetation (Zhang et al. 2019). Soil pH levels can significantly impact the acid stress response of plants, as acidic soils can reduce nutrient availability and increase metal toxicity, which can in turn affect the microbial community. Tan et al. (2020) stated that the microbial community structure was linked to soil pH and annual precipitation. To elucidate the predominant environmental determinants shaping bacterial community diversity and co-occurrence patterns in 21 maize field soils across China, an extensive investigation was carried out, entailing the utilization of high-throughput 16S rRNA gene sequencing coupled with meticulous network analysis. The principal objective was to identify and understand the principal influencers accounting for the observed heterogeneity within the microbial communities inhabiting these agricultural ecosystems. In order to assess the diversity and distribution of microbial communities, researchers utilized operational taxonomic units (OTUs) derived from the 16S sequencing data. OTUs were employed to classify and analyse microbial communities, representing clusters or groups of microorganisms with similar genetic characteristics. In addition, to comprehensively study and quantify microbial communities, various indices such as alpha diversity and beta diversity can be employed. Alpha diversity captures the richness and evenness within individual communities, while beta diversity elucidates the dissimilarities or similarities in microbial composition between different samples or habitats (Walters and Martiny 2020). Their results showed that the OTU richness of the soil microorganisms had a significant positive correlation with soil pH (5.23–8.86) (Tan et al. 2020) based on the alpha diversity index.

#### Temperature

The temperature of the environment is another important parameter that affects the acid stress response of microorganisms. Changes in the temperature can directly affect the physico-chemical properties of acidic environments, as well as the physiology and behaviour of organisms experiencing the acid stress (Price and Sowers 2004). Temperature can influence the processes involved in acid tolerance and resistance, and can impact the acid stress response of microorganisms in several ways affecting enzyme activity, membrane fluidity, metabolic rate, and protein stability (Beales 2004). For instance, elevated temperatures have been found to enhance the solubility of acidic pollutants and the release of protons from organic matter. A study by Binet et al. (2020) demonstrated the influence of global warming and acid atmospheric deposition on carbonate dissolution and CO_2_ fluxes within French karst hydro systems. To accurately assess the quantitative impact of global warming on the uptake of atmospheric/soil CO_2_ through carbonate weathering, they distinguished the amount of [Ca + Mg] in water caused by the breakdown of soil carbonic acid from the amount caused by strong acid pollution. Through this method, they revealed that the acid pollution inputs hinder the increase in [Mg] (Binet et al. 2020).

#### Nutrient availability

Another parameter that can impact the acid stress response of microorganisms in heterogeneous environments is the availability of nutrients. The absence of certain nutrients, mainly carbon, nitrogen, phosphorus, and sulphur, may hinder microorganisms’ ability to cope with acidic conditions (Guan and Liu 2020). Nutrient availability can influence the rate of growth and metabolism of microorganisms, which can in turn impact their ability to tolerate acidic conditions. A nutrient-poor environment may reduce the ability of microorganisms to produce an acid stress response (Shimizu 2013, Gottesman 2019). Besides macronutrients, micronutrients (e.g. trace elements) can also affect the survival of microorganisms under acidic conditions (Murdoch and Skaar 2022). Furthermore, the ability of microorganisms to tolerate acidic conditions is dependent on their ability to maintain a stable internal pH and protect cellular components from acid damage (Gottesman 2019). Some microorganisms can utilize acidic nutrients such as organic acids to buffer against further acidification, which can impact the production of these organic acids, that can in turn affect the ability of microorganisms to tolerate acidic conditions (Gottesman 2019, Guan and Liu 2020). Moreover, the competition for the limited nutrient resources in a community affects the growth and metabolism of microorganisms, which can influence their ability to tolerate acidic conditions (Hibbing et al. 2010).

Monitoring nutrient availability is thus important for comprehending ecosystem dynamics in various environments. In the context of soil ecosystems, numerous methods have been developed to assess nutrient composition. To evaluate the nutrient composition of soil, several approaches can be employed. The following methods are commonly utilized in soil nutrient analysis (Mallarino 2023, Marschner and Rengel 2011). Most of these methods are also applicable for analyzing nutrient availability in diverse ecosystems, including aquatic environments, as well as controlled systems such as bioreactors or constructed wetlands.

- Macronutrient analysis: macronutrient assessment entails determining the total nitrogen content using either the Kjeldahl digestion or Dumas combustion methods. Total phosphorus can be quantified using colorimetric techniques. Potassium levels can be measured using flame photometry or atomic absorption spectroscopy after extraction.
- Secondary and micronutrient analysis: secondary nutrients, including calcium, magnesium, and sulphur, can be quantified using atomic absorption spectroscopy or inductively coupled plasma methods. Similar methods can be used to quantify essential micronutrients such as iron, zinc, manganese, copper, boron, and molybdenum.
- pH and electrical conductivity measurements: pH can be determined using a pH meter, while electrical conductivity can be measured using a conductivity meter.
- Organic matter content assessment: evaluating the organic matter content in soil is crucial for understanding nutrient cycling and soil health. The loss-on-ignition (LOI) technique is widely used, wherein soil samples are subjected to high temperatures to estimate the percentage of organic carbon.

Besides abiotic factors, biotic factors such as genetic variability, metabolic capacity, and interactions of microorganisms within a community, have significant influence on the acid stress response of microorganisms (Braga et al. 2016, Guan and Liu 2020, Alnahhas and Dunlop 2023). Some microorganisms may have symbiotic relationships with other microorganisms that help them cope with acidic conditions, while the presence of predators or competitors may increase stress levels and reduce the ability of populations to adapt (Braga et al. 2016, Alnahhas and Dunlop 2023). Such relationships can be studied using coculture experiments. For example, Aranda-Diaz et al. (2020) cocultured *L. plantarum* with *Acetobacter* species to understand how *Acetobacter* species counter the acidification driven by the *L. plantarum* production of lactate. They showed that due to the pH shifts modulated by *L. plantarum*, inhibition by rifampin and erythromycin was well-tolerated. Furthermore, the researchers identified the interaction between *L. plantarum* and *Acetobacter* species by studying the changes in antibiotic tolerance, measuring key metabolites, and assessing environmental pH levels in synthetic communities constructed from the fruit fly gut microbiota. The identification of interspecies interactions in mixed communities and complex systems can provide insights into their response and tolerance to diverse environmental conditions, including acid stress response (Aranda-Díaz et al. 2020).

### Responses of heterologous populations and community profiling

Assessing the reactions of heterologous populations and community profiling in the face of acid stress can aid understanding of the diversity and functional capacity of microbial communities. Such measurements have the potential to provide valuable insights into the response mechanisms of these communities and their ability to thrive in acidic conditions. These methods mainly consist of culture-dependent and culture-independent techniques.

Culture-dependent methods, as described in the first sections of this review, rely on microorganisms being able to grow and replicate under certain conditions. However, acid stress can significantly inhibit the growth of many microorganisms, which can lead to inaccurate or unreliable results when using culture-dependent methods to measure the acid stress response. Culture-independent methods including ‘omics-based methods have the potential to provide a more comprehensive and precise description of the acid stress response of mixed populations of microorganisms. The following section outlines some of these.

#### Genomics in the study of microbial consortia

In addition to traditional methods for identification of acidophilic microorganisms (Zhang et al. 2013), nowadays more studies lean towards ‘omics methods for a more detailed description of the microbial community or individual culturable or unculturable microorganisms in acidic environments. By examining the genomes present in microbial communities (metagenomics), it is possible to describe the species composition of acidophilic communities, their ecological network (Quatrini and Johnson 2018a), and the phylogenetic relationship of the individual species (Johnson 2018).

The genomic study of environmental acidophilic consortia faces several challenges. A general point about metagenomics is that consortia typically consist of multiple interacting species, which can make it difficult to accurately separate and assemble the individual genomes within the complex community. Additionally, the acidic and extreme conditions in which these consortia thrive can result in rapid DNA degradation and contamination on extraction, further complicating genomic studies (Massello and Donati 2021). Overcoming these challenges requires the development of specialized techniques and bioinformatics tools to improve genome assembly, enable the analysis of complex microbial communities, and ensure the accuracy of genomic data obtained from acidophilic consortia

Methods using genetic information to study single species can be effectively applied to the study of bacterial communities in natural environments. To describe a bacterial community, 16S rRNA or functional gene amplicon sequencing, and whole shotgun metagenomic sequencing are the most preferable options (Duquesne et al. 2008, Schoch et al. 2012, Hua et al. 2015). We will discuss these in turn.

16S rRNA sequencing (not strictly genomics, but using the same methods) involves identifying and classifying bacteria by analyzing a specific region of the 16S ribosomal RNA gene. This highly conserved gene region is sequenced and compared to reference databases to identify the bacterial taxa in a sample (Celis et al. 2022). By sequencing the 16S rRNA gene of the bacteria within a consortium, it is possible to identify different bacterial species or OTUs, evaluate their relative abundance, and infer their potential roles and interactions within the consortia (Cárdenas et al. 2016). Several different pipelines for analysis of 16S rRNA sequence data exist, including Silva (Celis et al. 2022), Picrust (Langille et al. 2013), Tax4Fun (Abhauer et al. 2015), or PAPRICA (Bowman and Ducklow 2015). It is also possible to investigate the presence of eukaryotes with the use of 18S rRNA fragments (Zhang et al. 2021). Fungi are most often identified by analysing PCR-amplified internal transcribed spacer region of nuclear rRNA genes, however this method may give reliable information only up to the genus level and additional sequences are required for species based identification (Schoch et al. 2012, Hibbett et al. 2016). Based on this type of analysis, it was for example shown that core assemblages of acidophile communities consist of a single dominant species or a couple of codominant species generally constitute 50%–80% of the community (Quatrini and Johnson 2018a, Massello et al. 2020) and the diversity of the bacterial community decreases with increasing environmental acidity (Cowan et al. 2015, Quatrini and Johnson 2018a, Zhang et al. 2021). Among eukaryotic organisms, it is possible to find mainly photoautotrophic algae, such as *Chrysonebula sp., Coccomyxa sp*., and *Ochromonas sp*. (Zhang et al. 2021).

Another widely used method for describing acidophilic consortia is functional gene sequencing. The principle of functional (amplicon) gene sequencing involves selectively amplifying and sequencing a specific target gene or genes of interest. This method utilizes PCR to amplify the target gene region using gene-specific primers, followed by DNA sequencing. By targeting specific functional genes and using quantitative next-generation sequencing methods where the frequency of reads of a given sequence gives a measure of its abundance in the starting population, this approach allows the assessment of the presence, abundance, and diversity of genes associated with biological functions or processes within microbial community (Saluja and Sharma 2014, Yuan et al. 2022). This methodology has been applied to several types of acidophilic bacteria (Yuan et al. 2022), fungi (Nkuna et al. 2022), and algae (Hsieh et al. 2018) in order to describe genes that participate in bioleaching and biomining processes and processes of metal removal from the environment.

In comparison to other methods, whole metagenomic sequencing provides a wealth of additional information. This approach involves sequencing all the genetic material (DNA) present in the sample without prior targeting or amplification of specific genes (Goltsman et al. 2009). One of the key contributions of whole metagenomic sequencing in studying acidophilic consortia is the ability to identify genes related to the life of acidophilic bacteria in a community, such as quorum sensing (Farah et al. 2005) and biofilm formation (Barreto et al. 2005a,b, Bonnefoy 2007, Goltsman et al. 2009). Additionally, metagenomic data can be used to reconstruct draft genomes of individual microorganisms present in the acidophilic community, including rare species, facilitating the description of new acidophilic bacterial species. This approach enables the identification of novel genes and pathways and provides more detailed information on the structure of the microbial community (Cowan et al. 2015, Christel et al. 2018).

#### Transcriptomic analysis of microbial communities

In addition to providing information about the gene expression in individual organisms, transcriptomics has been used to study the response of microbial communities to different environmental conditions (Bonnefoy 2007, Cárdenas et al. 2010, 2016). By investigating gene content, expression patterns, and taxonomic composition, researchers can uncover the functional activities within acidophilic communities and specific levels of gene transcription associated with response and adaptation to different environmental conditions (Chen et al. 2015).

Meta-transcriptomics, involving sequencing and analyzing microbial community transcripts (mRNA), poses unique challenges in mixed acidophilic populations. These include low biomass, RNA degradation, incomplete rRNA removal, and low transcript capture efficiency for detecting low-abundance transcripts (Garrido et al. 2008). Overcoming these challenges involves careful sample collection and preservation, specialized RNA extraction methods (e.g. acid–phenol-based and RNA stabilization), customized rRNA depletion using acidophilic-specific sequences, and optimizing library preparation and sequencing to capture low-abundance transcripts (Lebuhn et al. 2016, Christel et al. 2018, Ayala-Munoz et al. 2020, Dopson et al. 2023).

DNA microarrays and RNA sequencing have both been used to study micro-organisms in heterogeneous communities, and provides information at both the genetic and the species level (Bonnefoy 2007, Moreno-Paz et al. 2010, Slyemi et al. 2013, Miao et al. 2020). Transcriptomic methods have allowed researchers to describe the activity and regulation of metabolic pathways in acidophilic biofilms, including Fe(II) and reduced inorganic sulphur compounds oxidation and nitrogen fixation (Bonnefoy 2007, Moreno-Paz et al. 2010). These methods have also provided insight into gene regulation mechanisms, including those using regulatory small RNAs, associated with CO dehydrogenase as a response to environmental changes (Goltsman et al. 2019). Additionally, quorum sensing genes and those related to mixed acid fermentation have been found to be upregulated in biofilm formation (Moreno-Paz et al. 2010, Yin et al. 2019).

Transcriptomic methods have also been used to determine the composition of metabolically active members of microbial communities and to describe the functional activity of microbial community members (Moreno-Paz et al. 2010, Goltsman et al. 2019). RNA-seq has allowed researchers to investigate both coding genes and noncoding RNAs, providing a more comprehensive understanding of microbial responses (Goltsman et al. 2019).

## The use of models to study acid stress

### Modelling acid stress: what are predictive models and why are they necessary?

Up to this point, we considered experimental methods that can be applied when micro-organisms can be grown in cultures, whether as pure cultures or as microbial consortia. However, it is often the case (particularly in food and drink microbiology) that we need information about microbiological growth and general behaviour under conditions that are not experimentally easy to study. We also may want to make our understanding more predictive. Mathematical models are valuable tools in such cases, as they can be used for evaluating microbial responses in diverse substrates, including food products, across a range of environmental conditions (Perez-Rodriguez and Valero 2013). To construct these models, researchers usually rely on empirical data, which involves deliberately introducing a microorganism into a substrate and observing its response over time under static or dynamic conditions of temperature, pH, and other factors that may affect microbial behaviour. After development and/or validation, these models can be incorporated into user-friendly software to predict microbial processes such as growth, survival/inactivation, transfer, and the likelihood of growth or death in response to various factors (Possas et al. 2022). Model predictions can assist in making informed decisions concerning food safety and quality, for product and process optimization, in the development of quantitative microbial risk assessments, among other applications. Furthermore, predictive modelling can save valuable time and resources by reducing the need for costly and time-consuming experimental studies.

**Figure 5.**
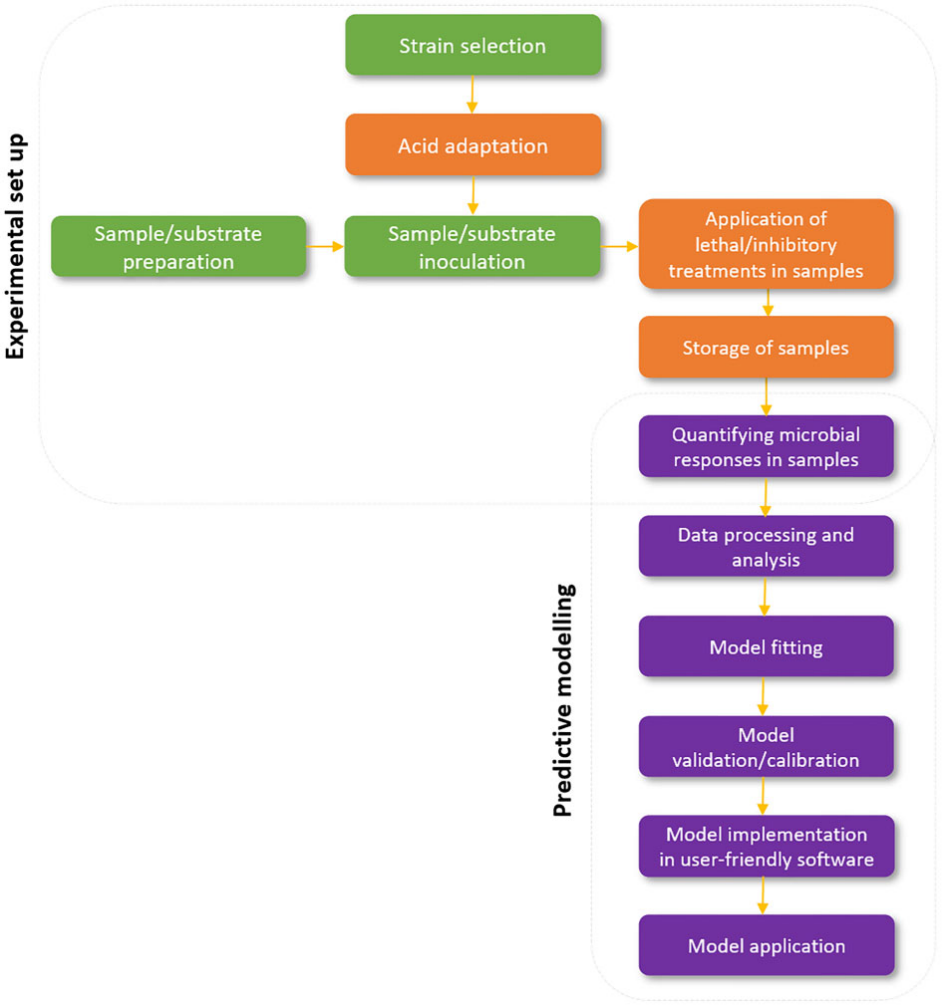
Flowchart showing the main steps for predictive microbiology model development. Orange boxes (light shading) represent nonmandatory steps, green boxes (medium shading) represent mandatory steps to obtain quantitative data, and purple boxes (dark shading) are essential steps for predictive modelling.

Mathematical models are instrumental in answering a wide range of questions, such as how changes in pH will impact the microbiological safety of a particular food product or how acid-adapted microbial cells will respond differently from nonadapted cells in a given medium. In the field of predictive microbiology, researchers have extensively applied these models to study the responses of acid-adapted cells, both *in vitro* and *in vivo*, to gain insights into their behaviour and better understand their impact on various systems (Skandamis et al. 2007). Estimation of microbial growth and inactivation parameters (e.g. maximum growth rates and inactivation rates) through modelling has been employed to compare microbial inter- and intraspecies variability with regards to acid resistance (Guillén et al. 2022). Modelling techniques have also been employed to assess whether a microbial population may exhibit induced acid stress resistance under conditions of dynamic pH and varying acid concentrations (De Jonge et al. 2003, Janssen et al. 2008). Additionally, modelling has been applied to assess how acid-shocked cells respond to other stresses, such as heat stress (Clemente-Carazo et al. 2021). These models are essential for evaluating the ability of microbial cells to survive under challenging conditions, such as the ones found in the human gastric acid barrier (De Jonge et al. 2003). The flowchart in Fig. 5 outlines the essential steps required to develop and implement a predictive model that considers the responses of acid-adapted or nonadapted cells in substrates with constant or dynamic pH values.

### Experimental set up methods

The experimental set up to obtain data for model development will depend on the specific goals of the study, as well as the nature of the processing environment being studied, and the modelling approach used. Many factors must also be considered when selecting microbial strains for modelling studies. A key concept is that of the Gamma hypothesis (Zwietering et al. 1992, Zwietering 2009), according to which microbial growth is influenced by different inhibitory factors that act independently (e.g. temperature, pH, and a_w_). To test this in growth modelling, it is preferable to use strains for which the cardinal parameters are known, such as the minimal, optimum, and maximum temperature, pH, and water activity (a_w_) required for growth (ISO 2019). For modelling purposes, the effect of these individual factors on microbial growth rate can be combined in a multiplicative manner in the form of ‘Gamma factors’ or ratios representing the observed growth relative to uninhibited growth (Zwietering et al. 1992). Previous knowledge about the minimal and optimal growth requirements greatly facilitates the calculation of each individual Gamma factor.

To account for the variability in microbial populations found in complex environments in modelling studies, such as those present in food processing facilities, researchers often use a multistrain cocktail for sample inoculation instead of relying on just one individual strain. Nonetheless, in order to compare the variability between different strains regarding growth potential or sensibility to lethal treatments, independent experiments must be carried out for each individual strain separately. Overall, when selecting strains to obtain data for model development, it is essential to meet certain basic requirements to assure experimental reproducibility. These include accurately identifying and characterizing the strains, making sure they are available in national or international culture collections, and knowing their original sources (ISO 2019).

Once the appropriate strain(s) have been selected, the methods for acid stress adaptation (preinduction) described earlier in this review can be utilized if the objective of the predictive model is to estimate the responses of acid-adapted cells or to compare the responses of acid-adapted and nonadapted cells. Overall, the addition of glucose (1%) to the growth broth prior to incubation is a simple and effective method for acid-adapting bacterial cells that has been applied in modelling studies (Skandamis et al. 2007, Mataragas et al. 2008). Another method for acid-adapting microbial cells for modelling studies is to lower the pH of the growth media using an inorganic acid such as hydrochloric acid prior to incubation (Desriac et al. 2013, Kim et al. 2020a, Clemente-Carazo et al. 2021).

Prior to inoculation, the substrates used to assess microbial behaviour (e.g. food samples, soil, water, and culture media) are prepared according to the purpose of the study. For assessing the effect of pH on microbial responses, the pH of substrate is usually modified by adding organic acids (e.g. lactic, citric, and acetic) and other acids such as hydrochloric. Afterward, batches or aliquots of the substrates can be divided into smaller sample units or, alternatively, suitable equipment, such as reactors or fermenters, can be employed for carrying out the tests. Thereupon, acid-adapted and/or nonadapted microbial cells are inoculated in the sample units or substrates. The adjusted initial microbial concentration inoculated will depend on the microbial process expected over time. More specifically, to characterize and model microbial growth, the initial concentration is usually set at values within the range 10^2^–10^3^ CFU/g or ml to allow an appropriate characterization of the microbial growth curve. On the other hand, for modelling microbial survival or inactivation, initial concentrations within the range 10^6^–10^7^ CFU/g or ml are preferred, so microbial reductions can be appropriately quantified.

For evaluating microbial responses, inoculated sample units are usually stored under conditions that are realistic representations of real-life scenarios (i.e. isothermal or dynamic temperature conditions, relative humidity, and aerobic or anaerobic conditions) for appropriate times. Depending on the substrate and on the inoculated microorganisms, fermentations may take place, reflecting processes in which the decrease on pH may have a significant impact on microbial responses (e.g. production of fermented foods such as cheeses) (Possas et al. 2021a). Samples are then periodically withdrawn at appropriate time intervals to be subjected to microbiological analysis to enumerate viable cells. Additionally, relevant physicochemical parameters of the samples, mainly pH and a_w_, are also monitored using appropriate instruments. Moreover, microbial growth experiments to obtain data for model development have been frequently carried out applying absorbance methods using spectrophotometers such as the Bioscreen (Lindqvist and Lindblad 2011). However, this method cannot be applied for complex and opaque matrices, like soils and food samples, only for transparent liquids, and does not allow pH monitoring during growth experiments. Another limitation of using spectrophotometers is that small changes in cell growth may not be detected by the instrument, due to its low detection limit.

Many modelling studies have focused on the evaluation of lethal treatments (e.g. heat treatment and high pressure processing) on microbial inactivation kinetics, while others have focused on modelling the effects of combining these treatments with low pHs to increase overall microbial reduction (Possas et al. 2021b). Furthermore, the inactivation kinetics of adapted and nonadapted cells exposed to acid lethal conditions have been modelled (Desriac et al. 2013). In studies on microbial inactivation, inoculated samples are subjected to lethal treatments that involve the application of specific environmental conditions such as different acid pH for varying exposure times. Microbial cells are quantified at different treatment exposure times to generate a dataset for model fitting. Additionally, the effect of these lethal treatments on the development of acid stress resistance has also been a topic for investigation in modelling studies (Guillén et al. 2022).

More information on all the aspects concerning the experimental set up to obtain data for the development of predictive models, including appropriate number of sample units, number of units that must be analysed at each time point can be found in ISO 20976–1:2019 (ISO 2019), and other relevant references (National Advisory Committee on Microbiological Criteria for Foods 2010, Perez-Rodriguez and Valero 2013).

### Modelling methods

The quantitative data obtained following the methods described in the experimental set-up section are processed and changes in microbial concentration are usually expressed as a function of storage or treatments. Model selection is made based on the available data, on the purpose of the study and on the desired level of complexity and accuracy in the predictions. Many user-friendly tools are available online for model fitting, which have greatly benefited users who may not have a strong mathematical background and struggle with complex equations. However, the computational intensity of these models can vary depending on factors such as the complexity of the model structure, number of parameters, type of variables (dynamic versus static) and the dataset size. Although user-friendly interfaces facilitate the model fitting process, users should be aware that more complex models or larger datasets may require significant computational resources.

Possas et al. (2022) have recently reviewed the predictive microbiology tools available for model fitting, including links to access each one of them. Examples of these tools include two Shiny applications in R: bioinactivation (Garre et al. 2018; https://github.com/albgarre/biogrowth) and biogrowth (Garre et al. 2023) for modelling microbial inactivation and growth, respectively. Briefly, to use these tools users must upload or manually insert growth or inactivation data (e.g. microbial concentration over time) and select a model among a list of mathematical models implemented into the software. Once a model is selected, model fitting is done. As results of model fitting, users get the estimates of model parameters, the graphical representation of the model and its goodness-of-fit indices. To evaluate the accuracy and suitability of different models to describe quantitative data, various statistical indices are used to compare observed and predicted data. These statistical indices include, but are not limited to, the adjusted coefficient of determination (*Rad j*²), log-likelihood value, and the root mean square error (RMSE). Based on these indices, confidence levels for estimated parameters and visual analysis, users must decide whether the selected model is suitable, and can compare the performance of different models to describe the quantitative data.

In the field of predictive modelling, different types of models are used. These include empirical and mechanistic models. Empirical models do not necessarily consider the mechanisms or physiological processes underlying microbial behaviour, but instead focus on the statistical patterns observed in the experimental data. Mechanistic models are based on an understanding of the underlying physiological or biochemical processes that govern microbial behaviour. These models are typically more complex and include parameters with biological significance. Each type of model has its own advantages and limitations, and the most appropriate model for a particular application will depend on the specific context and research objectives.

To evaluate microbial growth kinetics in foods, lag times, growth rates, and maximum population densities (i.e. growth kinetic parameters) are usually estimated by fitting the sigmoid Baranyi and Roberts, the Gompertz, and the Logistic models to microbial quantitative data. On the other hand, the Arrhenius-type, Weibull, Geeraerd, and Biphasic models, are the most applied for describing microbial inactivation kinetics in foods and estimating inactivation rates. In predictive microbiology terminology, these models, which describe changes in microbial concentrations over time, are known as primary models. Models that describe the dependence of the parameters of primary models (e.g. growth and inactivation rates) on environmental factors such as temperature, pH, and a_w_ are known as secondary models. Cardinal models based on the Gamma hypothesis are considered secondary models including parameters with biological significance, such as the minimal temperature, pH and a_w_ for growth for a given microorganism. Although being more complex compared to other model structures, cardinal models are usually preferred by risk assessors, due to their biological significance. The models developed by Pin et al. (2011) and Coroller et al. (2015) are examples of cardinal models including the effects of pH, a_w_, and temperature on *Salmonella* growth. The latter also includes the effect of dynamic lactic acid concentrations, produced by LAB during sausage fermentation. According to these models, when the combination of factors do not allow *Salmonella* growth, inactivation can be predicted using the Arrhenius (Pin et al. 2011) and the Weibull (Coroller et al. 2015) models.

Mathematical models establishing the relationship between environmental variables and a binary response variable (i.e. growth/no growth) are also commonly employed when evaluating microbial acid stress responses. This relationship is computed as the logit P function and the mathematical function is derived by using linear or logit regression methods. These models can help clarify the growth–no-growth interface as the combination of environmental factors that separate growth and nongrowth regions. Growth boundaries are influenced not only by the pH level but also by the type of acid used (McKellar and Lu 2001). Furthermore, the use of pH-adapted strains can significantly alter growth limits. For instance, it has been shown that pH-adapted *E. coli* O157:H7 can shift its growth limits towards lower pH values compared to nonadapted strains, while also increasing its growth tolerance to lower a_w_ values (Skandamis et al. 2007). In spore-forming bacteria, pH adaptation can also impact the probability of germination, as demonstrated in *Clostridium sporogenes*, which increased its germination probability in the pH range of 5.0–5.5, compared to nonadapted strains. This adaptation modifies the boundaries towards a combination of lower pH and higher NaCl (Valero et al. 2020).

In the last decades, effort has gone into the development of microbial interaction predictive models, which describe the dynamics of mixed microbial populations in a common environment. These models depict the simultaneous growth of bacterial foodborne pathogens with other microbial populations, such as *L. monocytogenes* and LAB (Guillier et al. 2008). To date, the Jameson-effect and the Lotka–Volterra models are the most applied to describe microbial competition. The Jameson-effect model is a simplified approach that assumes that LAB and the pathogen exhibit equal levels of inhibition towards each other, as they do towards their own growth (Jameson 1962). This means that the growth of one microorganism stops when the other reaches its maximum density. On the other hand, the Lotka–Volterra model assumes a prey–predator relationship between different microbial populations that can also be linked to the competition for a common nutrient or substrate (Vereecken et al. 2000). Neither the Jameson nor the Lotka–Volterra models are designed to capture individual cell-level dynamics, but rather they describe population-level interactions between two partners (Powell et al. 2004). In addition, they typically assume homogeneous microbial populations. If the research context involves more complex microbial communities with interactions among multiple microbial species, it would be necessary to explore alternative modelling approaches that can accommodate these complexities. Potential approaches could include extending the Lotka–Volterra framework to multispecies interactions or using more advanced modelling techniques capable of capturing interactions within diverse microbial consortia (e.g. network-based models) (Powell et al. 2004).

For model validation, additional independent experiments considering factors within the model domain are performed to evaluate whether model predictions are consistent with observed data. Additionally, data used for validation can be derived from independent published research or databases of microbial responses (e.g. ComBase available in www.combase.cc). Various statistical indices can be used for model validation, such as RMSE, standard error of prediction, accuracy and bias factors (Baranyi et al. 1999).

Predictive models have been implemented into user-friendly software to be used for a series of applications, such as on the processes optimization, risk assessments, and shelf-life estimation (Tenenhaus-Aziza and Ellouze 2015, Possas et al. 2022). Examples of applications including models that consider the effects of pH on microbial behaviour are the ComBase Premium (www.cbpremium.org), ComBase, MicroHibro (González et al. 2019), Food Safety and Spoilage Predictor (FSSP) (http://fssp.food.dtu.dk/), and Sym'Previus (Leporq et al. 2005). Among these, Sym'Previus include cardinal models for various microorganisms in culture broth, and allows to enter dynamic pH, a_w_, and temperature profiles to obtain estimations of microbial concentrations under these conditions. As well as allowing simulations under dynamic conditions of temperature, pH and a_w_, MicroHibro also allows the integration of the predictive models available on its database into quantitative microbiological risk assessments following a very intuitive structure (González et al. 2019). Developers of new or updated versions of these user-friendly tools should consider including microbial stress adaptation, such as acid adaptation, as a prediction parameter to estimate microbial behaviour in foods.

For model application to get estimates of microbial concentrations and kinetic parameters, users must access a tool online or download it, select a model, and define its input variables, such as temperature and pH. Alternatively, model equations can be implemented using other interfaces or programming resources, such as Excel and R. It is important to highlight that models must be applied within the domains of variables used for model development, including ranges of temperatures, pHs, and other relevant factors considered. Extrapolations to values outside the model domain may result in over or under estimations of microbial growth, inactivation and survival, or transfer. Finally, when applying a model, users must be aware of the limitations and uncertainties inherent to model simulations, to accurately interpret the results obtained.

### Limitations and future perspectives of predictive modelling

Predictive models have traditionally been developed using quantitative data obtained in culture media, in which the conditions can be easily standardized, and microbial responses are more reproducible with lower variability. The predictions of models developed in culture media have been extrapolated to other substrates, mainly foods. However, food matrices are typically very complex environments, where the structure of the food product can have a significant impact on the behaviour of microbial populations (Mataragas et al. 2008, Verheyen and Van Impe 2021). To overcome this problem, validation of these models developed in culture media prior to their application in other substrates is highly recommended. Additionally, it is essential to note that in many cases, models have been developed using individual or few microbial strains, not reflecting interstrain variability in terms of acid stress resistance. This limitation could potentially lead to over or underestimation of microbial growth, survival, or inactivation in different scenarios.

To persist at low pHs and other adverse conditions, some bacteria display adaptations such as changes in the duration of lag phase (Français et al. 2019). Modelling of lag times remains unsatisfactory due to uncertainty surrounding the timing of events that occur during this phase (Français et al. 2019). It was demonstrated in one study that the *Bacillus cereus cshA* gene could be applied as molecular marker, since its expression provides information on the events occurring during lag phase (Français et al. 2019). In another study, authors also evaluated gene expression as a molecular biomarker to predict microbial acid resistance (Desriac et al. 2015). These studies suggest that the combination of biomarkers with predictive microbiology models could be a strategy to better predict the physiological state of cells. In this respect, combining ‘omics with modelling would increase our prediction power, reducing the uncertainty when performing microbial risk assessments (den Besten et al. 2018). In this sense, it is expected that future studies within the domain of predictive modelling and risk assessments will include ‘omics results to obtain more realistic predictions of microbial behaviour.

## Conclusion

In this review, we have endeavoured to illustrate the wide range of different methods that can be used to study the effects of acid on micro-organisms, as well as drawing attention to some of their limitations. The use of these tools in different combinations means that we know a great deal about the ways in which many different micro-organisms respond to low pH, and how some of these responses are important for their growth and survival. We have methods to look at these at many different levels, with implications both for our understanding and exploitation of micro-organisms, whether as pure cultures or mixed consortia. But it is also true that we do not yet have a complete description, or understanding of, all the changes and their adaptive significance that occur at low pH in any single organism, let alone in a heterogeneous population or in mixed cultures in the laboratory or the natural environment.

Studies on acid stress are inherently complex, partly because the terms ‘acid’ and ‘low pH’ encompass a broad range of conditions, and partly because of the dependence of acid resistance, acid tolerance, and the acid stress response, on multiple biotic and abiotic factors. This means that any investigator working in the field needs to be aware of, and needs to report, the maximum amount of relevant information about their studies, so that they can be of maximum use in moving the field forward. Many high throughput fields now have minimum requirements for data deposition in online databases, and we suggest that similar requirements should apply in the field we have reviewed here. Although much of the literature in the field is of very high quality in terms of how experiments are described, sometimes key details are omitted that can make experiments impossible to reproduce. As a minimum, authors should report the full conditions of any experiment, including:

- all constituents of all media used, including buffers, whether these are used for growth, stress, killing, or recovery;
- the pH of all media and which acids were used to adjust the pH;
- whether the pH changed during the course of the experiment;
- the length of time for which organisms have been grown and under precisely what conditions including container size and shaking speed (aeration can have a significant impact on acid resistance and acid tolerance);
- the state of growth of the cells (as many authors use expressions such as ‘mid-logarithmic phase’ rather loosely; ideally, growth curves should be included in supplementary data).

The specific organism studied should be described in as much detail as possible: changes of a single base at the genome level can have very major effects on responses to low pH (Eguchi and Utsumi 2014), so in cases where whole genome sequence data are available they should be referred to. Authors need to be aware too of strain heterogeneity (the most used strain of the most used model organism, *E. coli* MG1655 K12, is known to vary between laboratories (Freddolino et al. 2012) and be cautious about making extrapolations from data obtained on laboratory strains.

None of these are reasons for not working in what is an exciting and relevant field of microbiology, and we hope that reading this review may stimulate new people to consider joining the global community who are working on all aspects of acid resistance, acid tolerance, and acid stress responses in micro-organisms.
